# Maternal behavioral thermoregulation facilitated evolutionary transitions from egg laying to live birth

**DOI:** 10.1093/evlett/qrad031

**Published:** 2023-07-26

**Authors:** Amanda K Pettersen, Nathalie Feiner, Daniel W A Noble, Geoffrey M While, Tobias Uller, Charlie K Cornwallis

**Affiliations:** Department of Biology, Lund University, Lund, Sweden; School of Life and Environmental Sciences, The University of Sydney, Sydney, Australia; Department of Biology, Lund University, Lund, Sweden; Division of Ecology and Evolution, Research School of Biology, The Australian National University, Canberra, Australia; School of Natural Sciences, University of Tasmania, Sandy Bay, Australia; Department of Biology, Lund University, Lund, Sweden; Department of Biology, Lund University, Lund, Sweden

**Keywords:** embryo, oviparity, plasticity, reproductive mode, squamate, viviparity

## Abstract

Live birth is a key innovation that has evolved from egg-laying ancestors over 100 times in reptiles. However, egg-laying lizards and snakes can have preferred body temperatures that are lethal to developing embryos, which should select against prolonged egg retention. Here, we demonstrate that thermal mismatches between mothers and offspring are widespread across the squamate phylogeny. This mismatch is resolved by gravid females adjusting their body temperature towards the thermal optimum of their embryos. We find that the same response occurs in both live-bearing and egg-laying species, despite the latter only retaining embryos during the early stages of development. Importantly, phylogenetic reconstructions suggest this thermoregulatory behavior in gravid females evolved in egg-laying species prior to the evolution of live birth. Maternal thermoregulatory behavior, therefore, bypasses the constraints imposed by a slowly evolving thermal physiology and has likely been a key facilitator in the repeated transition to live birth.

## Introduction

The evolution of live birth is an important life-history adaptation in vertebrates (Doody & Moore, 2010; Lambert & Wiens, 2013; Shine, 1985). The ecological conditions that favor the transition from egg-laying (oviparity) to live birth (viviparity) are relatively well understood, especially in reptiles, with particularly strong support for the adaptive value of viviparity in cool climates (Lambert & Wiens, 2013; Shine, 1995; Tinkle & Gibbons, 1977). Oviparous lizards and snakes typically lay eggs just after the first trimester of embryonic development, when the embryos develop limb buds (Andrews & Mathies, 2000; Shine, 1983). By retaining embryos until development has been completed, viviparous mothers can buffer offspring from suboptimal nest temperatures, ensuring faster development, higher hatching success, and increased offspring viability (Beuchat, 1988; Le Henanff et al., 2013; Shine, 1995; Warner & Shine, 2007). This transition has allowed reptile species to persist and diversify in cool climates across the globe (Ma et al., 2018).

Despite the clear adaptive advantages of live birth in some contexts, the evolutionary transition from oviparous ancestors to viviparity is challenging to explain. Evidence from case studies of lizards and snakes show that embryos and adults of oviparous species have different thermal requirements, with adult-preferred body temperatures often exceeding the upper lethal limit of embryos (Beuchat, 1988; Galliard et al., 2003; Mathies & Andrews, 1997; Qu et al., 2011). For example, the average nest temperature of the Iberian emerald lizard, *Lacerta schreiberi*, is 24°C, rarely exceeding 30°C, whereas the preferred body temperature of females is 33°C (Monasterio et al., 2013). Since embryos are well adapted to the temperatures they typically experience in the nest, they generally have limited capacity to develop at temperatures outside of this range (Du et al., 2019; Noble et al., 2018b; Sanger et al., 2018). Therefore, if females retain eggs throughout development, embryos would experience prolonged exposure to temperatures that result in offspring malformations or even death (Andrews et al., 2000; Braña & Ji, 2000; Noble et al., 2018b; Van Damme et al., 1992).

A mismatch between thermal optima of embryos and adult females should select against prolonged embryo retention, inhibiting evolutionary transitions to live birth (Beuchat, 1988). Despite this apparent constraint, live birth has evolved over 100 times in squamate reptiles (Blackburn, 2006, 2015). How can we reconcile the repeated evolution of viviparity with the potentially widespread thermal mismatches between embryos and adults in oviparous species? One hypothesis is that when substantial mismatches in thermal preferences exist, viviparous females behaviorally adjust their body temperature when pregnant to close the gap between adult and embryo thermal optima. Such plasticity may temporarily come at a cost to female performance but may promote embryo growth and survival and eliminate the thermal barriers to the evolution of viviparity. An alternative hypothesis is that females do not adjust their body temperature when gravid, and instead, viviparity only evolves from oviparous lineages where adult and embryo thermal optima are aligned to begin with (e.g., there is no initial mismatch in thermal optima).

Here we examine how the evolution of live birth has been influenced by the capacity of females to adjust their body temperature when gravid and the sensitivity of embryonic development to temperature across squamate reptiles using phylogenetic comparative analyses. Data were extracted from the literature on reproductive mode (viviparous versus oviparous, *N*_species_ = 7,830), the preferred body temperature of (non-gravid) females (*N*_species_ = 163), the optimum temperature for embryos as measured by hatching success (*N*_species_ = 52), and the adjustment of body temperature by gravid females (*N*_species_ = 52) (Supplementary Tables S1 and S2). We first quantify the mismatches between adult and embryo thermal optima. Second, we test if adult females adjust their body temperature when gravid to better match the temperature optimum of their developing embryos. Third, we test whether this behavioral plasticity was more pronounced in viviparous species compared to oviparous females, as expected if thermal conflicts are more severe when embryos are retained throughout development in viviparous species. Fourth, using ancestral reconstructions, we test if viviparity more frequently evolves from ancestors where females have a greater capacity to adjust their body temperature, or if viviparity predominantly evolves in lineages where adult and embryo optima are aligned. Data were analyzed using Bayesian phylogenetic mixed models (BPMMs) that permit missing data, which was necessary because not all traits were measured for all species (for full details of the extent of non-overlapping data see Supplementary Tables S1 and S2).

## Methods

### Data collection

Data on reproductive mode were extracted from Pyron and Burbrink (2014). Information for all other variables was obtained through literature searches across three datasets using ISI *Web of Science* (v.5.30). Search results were imported and sorted for relevance using Rayyan software (Ouzzani et al., 2016). The results of literature searches are presented following the preferred reporting items for systematic reviews and meta-analyses (PRISMA) statement (Moher et al., 2009) in Supplementary Figures S1, S2, and S4. The final dataset used for analyses is presented in Supplementary Table S1.

#### *The preferred female body temperature (P*_*bt*_ )

To investigate the phylogenetic distribution of thermal mismatch, we collected data on the preferred body temperature of adult, non-gravid, females (*P*_*bt*_) for as many species as possible using search terms “body temperature*,” along with one of the following: “squamat*,” “lizard*,” and “snake*” which yielded a total of 1,075 papers. We only used data from studies that explicitly stated body temperatures were from non-gravid females (unless pooled male/female data stated no significant effect of sex) or were collected from outside the reproductive season. We additionally cross-referenced this search with articles cited in Sinervo et al. (2010), supplementing our original dataset with 42 studies (PRISMA statement; Supplementary Figure S1). This provided a final dataset of 163 species (*N*_viviparous species_ = 61, *N*_oviparous species_ = 103). Note that data for *Zootoca vivipara* was available for both viviparous and oviparous lineages and data for both reproductive modes were included in the analyses.

#### *The optimum temperature for embryo development (T*_*opt*_ )

To estimate the optimal temperature for embryo development for as many species as possible, we extracted information on how temperature impacts hatching success from the Reptile Development Database (RepDevo vers 1.0.2; (Noble et al., 2018a)), and from the literature using the search terms “temperature* AND incubat* AND hatch* OR surv*” along with one of the following: “squamat*,” “lizard*,”and “snake*,” which yielded a total of 671 papers (PRISMA statement; Supplementary Figure S2). We only included studies where three or more constant temperature treatments were used under controlled laboratory conditions, resulting in 661 papers being rejected due to irrelevance or overlap with the Reptile Development Database. The final *T*_*opt*_ dataset consisted of 51 species from 81 studies.

#### The adjustment of body temperature by gravid females (Hedges’ g)

We searched for published articles presenting data that directly compared the preferred body temperature of gravid (*P*_*bt-g*_) versus non-gravid (*P*_*bt*-*ng*_) adult female squamate species. The “title,” “abstract,” or “keywords” were searched with the terms “body temperature* AND gravid* OR reproduct*,” along with one of the following terms: “squamat*,” “lizard*,” and “snake*.” This yielded a total of 721 papers of which 648 papers were rejected due to irrelevance (PRISMA statement; Supplementary Figure S4). For this dataset, we only included studies that provided both sample size and error around mean preferred body temperature. Studies included laboratory experiments that used artificial temperature gradients (*n* = 37) as well as field studies that measured preferred basking temperatures (*n* = 36). Laboratory studies generally measured the body temperature of the same female during gestation (*P*_*bt-g*_) and either before or after gestation (*P*_*bt-ng*_) as repeated measures. In contrast, field studies often measured body temperature in a population during the reproductive season, comparing the body temperatures of gravid and non-gravid females at a single time point. Combining laboratory and field studies yielded a total of 73 studies published up to July 2022 from which effect sizes were calculated for 52 species (live bearing: *n* = 32 and egg laying: *n* = 20).

An effect size of the adjustment of body temperature by gravid females for each species was calculated as the standardized mean difference (Hedges’ *g*) in preferred body temperatures between non-gravid and gravid females (*P*_*bt-g*_ − *P*_*bt-ng*_) estimated with heteroscedastic population variances and adjusted for small sample sizes (Borenstein et al., 2009). Effect sizes were calculated using the “escalc” function with the setting “SMDH” in the R package *metafor* (Viechtbauer, 2010).

### Phylogeny

We used a recent phylogeny of squamates (Tonini et al., 2016). All analyses were repeated across a sample of 1,000 trees to account for phylogenetic uncertainty. For figures, we used the maximum clade credibility tree provided by Tonini et al. (2016).

### Statistical analyses

1. Estimating thermal optima of embryos

The thermal optima of embryos (*T*_*opt*_) was measured by estimating linear and quadratic relationships between incubation temperature and hatching success using a Bayesian phylogenetic mixed effects model (BPMM) with Markov chain Monte Carlo estimation fitted in the R package “*MCMCglmm*” (assumes a Brownian motion mode of evolution (Hadfield, 2010)). We modeled the proportion of eggs hatched (binomial error distribution) with linear and quadratic temperature effects as fixed effects and random intercepts and slopes for both linear and quadratic terms at the phylogenetic level. More specifically, we fit the following model:


$$\begin{array}{l} y_{i,j}=\;\left(u_{0}+\;u_{sp_{i}}\right)+\;\left(\beta_{f}+\;\beta_{sp_{i}}\right)*T_{i,j}+\;\left(\gamma_{f}+\;\gamma_{sp_{i}}\right)*{T_{i,j}}^{2}+e_{i,j} \end{array}$$

Where $y_{i,j}$ is the hatching success for species, *i*, at temperature (*T*) *j*; $u_{0}$ is the intercept and $u_{sp_{i}}$ is the species-specific random intercept effect for species, *i*; $\beta_{f}$ is the linear fixed effect of temperature on hatching success, whereas $\beta_{sp_{i}}$ is the random linear slope for species, *i*; $\gamma_{f}$ is the quadratic fixed effect for temperature and $\gamma_{sp_{i}}$ is the species-specific random quadratic slope. The random effects were assumed to follow a multivariate normal distribution with a mean of 0 and a covariance matrix based on the phylogenetic covariance matrix derived from the phylogenetic tree. Given that we fit an unstructured covariance matrix this allowed linear and quadratic terms to be correlated. The precision of *T*_*opt*_ estimates was variable because the number of temperatures that hatching success was measured differed across studies (mean number of temperatures per species ± SD: 7.62 ± 4.29, Supplementary Figure S3). Raw thermal performance data are provided in Supplementary Figure S3. Given that hatching success had a high phylogenetic signature (phylogenetic heritability (*H*^2^) = 56%, 95% CI: 35%–76%), we modeled thermal reaction norms at the phylogenetic level to help improve estimates of thermal optima for species with sparse raw data. We found that using phylogenetic models, as opposed to linear mixed models without phylogenetic information, allowed the *T*_*opt*_ of each species to be estimated with greater precision: *T*_*opt*_ estimates produced by phylogenetic models (BPMM) showed smaller sampling error and reduced convergence problems compared to non-phylogenetic models. The model was run for 1,100,000 iterations with a burn-in of 100,000 iterations and thinning rate of 500, leaving us with 2,000 samples from the posterior distribution. The convergence of models was examined as outlined in the section “*Model convergence and parameter estimation*.”

From our BPMM we estimated *T*_*opt*_, and its corresponding sampling variance, using the posterior distribution of fixed effects and the posterior distribution of species-specific random slopes (linear and quadratic) as follows:


$$\begin{array}{l} T_{opt}=\;-\;\frac{\left(\beta_{f}+\;\beta_{sp}\right)}{2\left(\gamma_{f}+\;\gamma_{sp}\right)} \end{array}$$

Where $\beta_{f}$ and $\gamma_{f}\;$ are the posterior distributions for linear and quadratic fixed effect estimates for temperature and $\beta_{sp}$ and $\gamma_{sp}$ are the posterior distribution for a given species-specific random effect extracted from the phylogenetic random slopes. Calculating *T*_*opt*_ using the posterior distribution of fixed and random effects meant that sampling error for a given species could be propagated through subsequent analyses (see below).

1. Quantifying the mismatch in thermal optima between adult females and embryos

To quantify the mismatch between the thermal optima of adult non-gravid females and embryos we used a multi-response Bayesian phylogenetic mixed effects models (MR-BPMMs) with *P*_*bt*_ and *T*_*opt*_ as Gaussian response variables implemented BPMMs in *R* with the *MCMCglmm* package (Hadfield, 2010). Separate intercepts were fitted for each response variable allowing the mean of *P*_*bt*_ and *T*_*opt*_ to be estimated. Unstructured phylogenetic and residual variance-(co)variance matrices were fitted for random effects to estimate variances for each trait and their covariances (Hadfield, 2010). The phylogenetic signature for each trait was calculated as the variance explained by phylogeny relative to total random effect variance (equivalent to heritability, *H*^2^, in the terminology of *MCMCglmm*). Phylogenetic and residual correlations between *P*_*bt*_ and *T*_*opt*_ were also calculated (Supplementary Table S3, Online repository R code “model M1.1”).

The accuracy of measures of *P*_*bt*_ and *T*_*opt*_ varied across species due to study design and sample sizes which can be accounted for by weighting data points by their inverse sampling variance using the “mev” term in *MCMCglmm*. However, missing values in sampling variances are not permitted in *MCMCglmm.* As data on the error and sample size were missing for *P*_*bt*_ and *T*_*opt*_, it would not have been possible to account for sampling error in our analyses without drastically reducing the size of our dataset. Consequently, we used multiple imputations with predictive mean matching in the *mice* package in R to impute missing errors and sample sizes (Buuren & Groothuis-Oudshoorn, 2011). Including phylogeny was not possible in our *mice* imputations, but we did not (*a priori*) expect missing error and sample size to have a phylogenetic signature. While we could have used the sampling variance for *T*_*opt*_ based on the posterior distribution, we chose to use *mice* so that it was consistent across all variables where sampling error needed to be imputed.

To incorporate uncertainty in imputations, 20 complete datasets were generated, and models were run across each dataset to create 1,500 sampling events (75 per dataset). Each sampling event consisted of 2,000 iterations with only the last iteration being saved. Estimates from the last iteration of each sampling event *i* were used as the starting parameter values for the next *i* + 1. To simultaneously account for uncertainty in phylogenetic relationships, we used a new phylogenetic tree for each sampling event (1,500 trees sampled). This led to a posterior sample of 1,500 iterations, the first 500 iterations were discarded as a burn-in and the remaining 1,000 were used to estimate parameters.

1. Testing if females adjust their temperature when gravid (Hedges’ *g*) in relation to potential thermal conflicts

We tested the prediction that females with high preferred body temperatures (*P*_*bt*_) downregulate their body temperature (negative Hedges’ *g*) when gravid, and vice versa, by calculating the phylogenetic correlation between *P*_*bt*_ and Hedges’ *g* (Supplementary Table S4, R code “model M2.1”). To do this, we used an MR-BPMM with *P*_*bt*_ and Hedges’ *g* as Gaussian response variables.

1. Testing if the adjustment of female temperature is more pronounced in viviparous species

To test if viviparous species adjusted their body temperature when gravid to a greater extent than oviparous species, we used a BPMM with Hedges’ *g* as a Gaussian response variable and reproductive mode as a fixed effect (Supplementary Table S5, R code “model M3.1”). Given that eggs are retained by females for longer periods in viviparous species compared to oviparous species, we may also expect a stronger effect of preferred body temperature on temperature adjustment in viviparous females (i.e., stronger correlation between Hedges’ *g* and *P*_*bt*_). To examine this possibility we used a MR-BPMM to test if the phylogenetic correlation between Hedges’ *g* and *P*_*bt*_ differed between oviparous and viviparous species. Reproductive mode was included as a fixed effect and separate unstructured phylogenetic and residual covariance matrices fitted for each reproductive mode specified using the “at.level” function in *MCMCglmm* (Supplementary Table S6, R code “model M4.1”).

1. Testing the effect of maternal plasticity and the alignment of embryo and adult thermal optima on the evolution of viviparity

To test if the evolution of viviparity is associated with the capacity of females in ancestral lineages to adjust their body temperature when gravid, and with the alignment of adult and embryo thermal optima, we used a two-step approach. First, the ancestral states of reproductive mode were estimated for each node in each of the 1,000 post-burnin trees. Second, we reconstructed ancestral values of Hedges’ *g* and the discrepancy between *P*_*bt*_ and *T*_*opt*_ for each node in each of the 1,000 trees and related this to whether nodes were involved in transitions to viviparity.

In the first step, we used hidden Markov models (HMMs) to reconstruct ancestral states of viviparity implemented in the R package “corHMM.” Previous studies have highlighted that the rate of evolution of viviparity varies across squamates and not accounting for such variation can lead to inaccurate ancestral reconstructions (Beaulieu & O’Meara, 2014; King & Lee, 2015; Wright et al., 2015) (King & Lee, 2015; Lee & Shine, 1998; Pyron & Burbrink, 2015). HMMs allow variation in the rate of binary character evolution to be estimated by predefining a number of rate categories from one state (e.g., oviparity) to another (e.g., viviparity) (Beaulieu et al., 2022). The most likely number of rate categories can be identified by comparing Akaike information criterion (AIC) values across models with different numbers of pre-defined rate categories. We found on the trimmed phylogeny (224 species) that AIC values were lowest when there were two rate categories (see R script “4. Models.R section 4”). This indicated that in two clades, transitions to viviparity occurred at a higher rate than in other parts of the phylogeny (Supplementary Figure S5). This model produced ancestral estimates that are consistent with the predominant view of viviparity evolution across squamates (Blackburn, 2015; King & Lee, 2015): a root state of oviparity and relatively few reversals of viviparity to oviparity compared to the transitions from oviparity to viviparity (Supplementary Table S7).

Estimates of ancestral states from HMMs were used to categorize transitions between oviparity and viviparity for each node in the following way: (1) oviparous with only oviparous descendants (oviparous to oviparous); (2) viviparous with only viviparous descendants (viviparous to viviparous); (3) oviparous with at least one viviparous descendant (oviparous to viviparous); and (4) viviparous with at least one oviparous descendant (viviparous to oviparous). To account for phylogenetic uncertainty, transition categories for each node were estimated across 1,000 trees (Supplementary Table S7). The predicted transition category for nodes can vary across trees due to differences in topology and internal tree structure. We, therefore, classified each node according to the most frequently predicted transition category.

In the second step, a MR-BPMM with Hedges’ *g*, *P*_*bt*_, and *T*_*opt*_ as Gaussian response variables was used to estimate ancestral values of each trait (R code “model M5.1”). Unstructured phylogenetic and residual variance-(co)variance matrices were fitted as random effects. The posterior distributions of predicted values for each node from this model were used to calculate mismatches in the thermal optima of females and embryos (CI of posterior distribution of *P*_*bt*_*– T*_*opt*_ not overlapping 0) and values of Hedges’ *g* (Supplementary Table S8). We also tested if the adjustment of female body temperature differed between the ancestors of oviparous and viviparous lineages by calculating if Hedges’ *g* was different from 0 (CIs not overlapping 0) for transitions from “oviparous to viviparous” compared to transitions from “oviparous to oviparous.”

Finally, we examined if viviparity evolves more frequently in lineages where the thermal optima of adults and embryos was aligned (Supplementary Table S9) by testing if oviparous nodes with similar thermal optima (CI of *P*_*bt*_ − *T*_*opt*_ overlapping 0) produced more descendent viviparous lineages than nodes where there were mismatches in thermal optima (CI of *P*_*bt*_ − *T*_*opt*_ not overlapping 0). Differences in frequencies were tested using a χ^2^ test of the number of nodes with and without thermal mismatches for oviparous nodes with oviparous descendants versus oviparous nodes with viviparous descendants (R script “5. Model_processing.R section 4”).

#### Prior specification

For BPMMs, we used non-informative uniform priors for fixed effects (*MCMCglmm* defaults) and a weakly informative inverse-Gamma prior for random effects (*V* = diag(*n*), ν = *n* − 1 + 0.002, where *n* was equivalent to the number of response traits). The sensitivity of parameter estimates to prior specification was examined by running single and multi-response BPMMs of Hedges’ *g*, *T*_*opt*_, and *P*_*bt*_ with parameter expanded priors (*V* = diag(*n*), ν = *n* − 1, alpha.mu = rep(0, *n*), alpha.*V* = 1,000), which have a lower pull toward zero, and by varying values of the shape parameter (single-response models: ν = 1, 2, 3; multi-response models: ν = (*n* − 1), 2(*n* − 1), 3(*n* − 1)). The influence of the priors on parameter estimation was checked by examining the overlap of trace plots and Gelman and Rubin’s diagnostic across models with different prior specifications (Brooks & Gelman, 1998). All models produced extremely similar estimates, suggesting the choice of prior had little influence on our results (see R script “4. Models.R” for details).

#### Model convergence and parameter estimation

Model convergence was assessed by running three independent MCMC chains and examining autocorrelation, which was low (lag values < 0.1), overlap in trace plots, which showed chains mixed well, and Gelman and Rubin’s convergence diagnostic that showed models converged (potential scale reduction factors were all below 1.1: R function gelman.diag (Brooks & Gelman, 1998)). Posterior distributions of all parameters were characterized using posterior modes and 95% credible intervals (CIs). Effects were regarded as significant where CIs did not span 0. *pMCMC* (number of iterations above or below 0/ total number of iterations) are also presented to facilitate general interpretation.

#### Verification analyses

##### Checking for differences in Hedges’ *g* between laboratory and field studies

Whether or not laboratory and field studies differed in their estimates of female adjustment of body temperature when gravid was checked using a BPMM with Hedges’ *g* as a Gaussian response variable, and study type (laboratory versus field) as a fixed effect (R code “model M5.4”; Supplementary Table S10). There were repeated measurements for some species so both species and phylogeny were fitted as random effects. We found that there were no significant differences in Hedges’ *g* between laboratory and field studies for either oviparous (0.17, CI = −0.54, 1.15) or viviparous species (−0.51, CI = −1.11, 0.11; Supplementary Table S10).

##### Checking robustness of results to missing data

BPMMs permit missing data in response variables, which was crucial given the patchy distribution of the data (Supplementary Table S1). The accuracy with which missing data is predicted is related to the phylogenetic signature in traits and the strength of phylogenetic correlations between traits (Molina-Venegas et al., 2018). All traits had high phylogenetic signature (phylogenetic *H*^2^ > 70%), hence there was high correspondence between raw data and predicted values from BPMMs (Supplementary Figure S6). We also tested how well models predicted ancestral values of reproductive mode with different degrees of missing tip data using HMMs in two ways. First, we compared the ancestral states of nodes predicted using all available data on reproductive mode from Pyron and Burbrink (2014) (*n*_species_ = 7,831, Supplementary Table S2) to the predicted states obtained using only the trimmed tree and data (*n*_species_ = 224). Second, we examined the accuracy with which ancestral nodes could be predicted on the phylogeny of 7,831 species using only reproductive mode data from the 224 species with thermal data. We found high correspondence between the ancestral states estimated using the missing and the full data (78% of nodes were predicted to be in the same state). The predicted ancestral states from both these HMM analyses can be found in Supplementary Table S11.

##### Checking the robustness of results to rate shifts

To verify that our ancestral estimates of Hedges’ *g*, *P*_*bt*_, and *T*_*opt*_ from the MR-BPMM were robust to variation in rates of evolution across the phylogeny we used phylogenetic ridge regression (PRR) implemented in the R package “RRphylo” (Castiglione et al., 2018). Phylogenetic ridge regression identifies shifts in continuous variables across a phylogeny by examining if mean differences between branches are greater than expected by chance using randomizations. We found that PRR models incorporating rate variation produced similar estimates to BPMMs for each trait (Pearson’s correlation coefficient (*r*): Hedges’ *g* = 0.79, *P*_*bt*_ = 0.96, *T*_*opt*_ = 0.98. R script “4. Models.R section 5”). Given rate shifts had minimal impact on the estimates of ancestral states, we used estimates from the MR-BPMMs because they: (1) allowed missing data; (2) incorporated sampling variances associated with response variables; (3) enabled phylogenetic correlations to be estimated; and (4) produced distributions of estimates (posterior samples) for each node that allowed significant thermal mismatches between embryos and adults to be calculated.

All analyses were conducted in R version 4.0.1 (R Core Team, 2020). References for all data collated can be found in Supplementary Table S12.

## Results

### The constraints imposed by thermal physiology

Across 52 species of reptiles (*N*_viviparous species_ = 32, *N*_oviparous species_ = 20), mismatches between the preferred temperatures of non-gravid females (*P*_*bt*_) and embryos (*T*_*opt*_) were widespread. On average *P*_*bt*_ of adult females was 4°C higher than *T*_*opt*_ (raw data mean ± SD: oviparous species, *P*_*bt*_ = 32.41 ± 4.15°C, *N*_species_ = 103; *T*_*opt*_ = 27.15 ± 1.92°C, *N*_species_ = 47; viviparous species, *P*_*bt*_ = 29.5 ± 4.06°C, *N*_species_ = 61; *T*_*opt*_ = 26.0 ± 2.23°C, *N*_species_ = 5). Incubating embryos throughout development at a temperature equivalent to their mothers’ *P*_*bt*_ was predicted to reduce hatching success by ~50% on average (Supplementary Table S1). This discrepancy may impose a constraint on the evolution of prolonged embryo retention. First, we found little evidence that the thermal optima of embryos (*T*_*opt*_) positively coevolves with the preferred body temperatures of non-gravid females (phylogenetic correlation (MR-BPMM): PM = 0.59, CI: -0.33, 0.93, *pMCMC* = 0.15; Supplementary Table S3). Second, both *P*_*bt*_ and *T*_*opt*_ were estimated to evolve slowly (MR-BPMM: *P*_*bt*_ phylo *H*^2^: 0.90, CI: 0.80, 0.96. *T*_*opt*_ phylo *H*^2^: 0.94, CI: 0.72, 0.99; Supplementary Table S3).

### Gravid females shift their body temperatures toward embryo thermal optima regardless of parity mode

**Figure 1.**
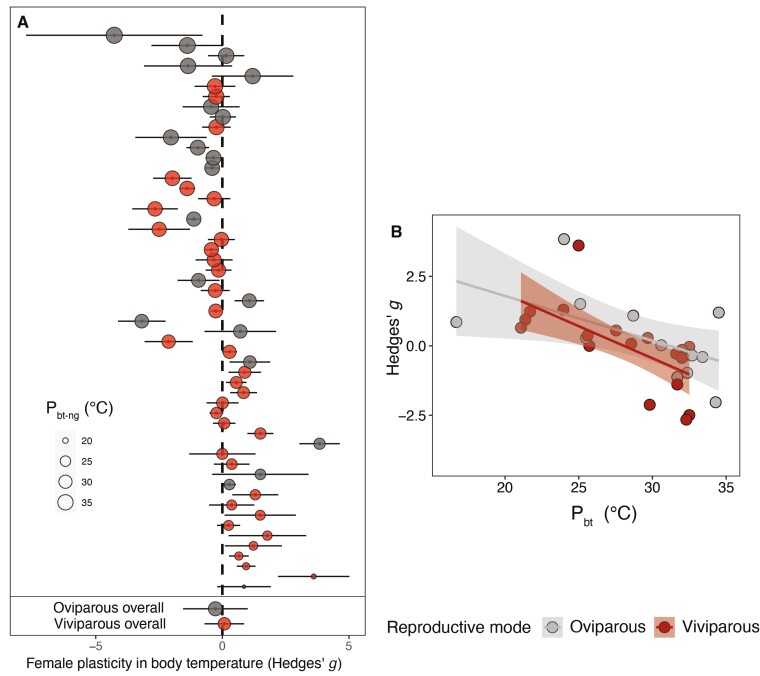
Plasticity in female body temperatures when gravid (Hedges’ *g*) resolves thermal mismatches between adults and embryos across 52 extant oviparous (*n* = 20) and viviparous (*n* = 32) squamate reptiles. (A) Adjustment of body temperature in gravid females (*P*_*bt-g*_) in relation to their non-gravid preferred body temperature (*P*_*bt-ng*_); Hedges’ *g*; *P*_*bt-g*_ − *P*_*bt-ng*_. Data points are ordered along the y axis according to *P*_*bt-ng*_. Points represent species means ± SEs and the size of points is scaled to indicate *P*_*bt-ng*_ (°C). (B) Relationship between Hedges’ *g* and *P*_*bt*_. Regression lines ± 95% confidence intervals are plotted. Positive values of Hedges’ *g* indicate higher gravid (*P*_*bt-g*_) versus non-gravid (*P*_*bt-ng*_) and negative values indicate reduced *P*_*bt-ng*_ when gravid (*P*_*bt-g*_) compared with non-gravid (*P*_*bt-ng*_). The plot shows that species with high *P*_*bt-ng*_ tend to reduce their body temperature when gravid (negative Hedges’ *g*), whereas species with low *P*_*bt-ng*_ tend to increase their body temperature when gravid (positive Hedges’ *g*).

We found that females significantly altered their body temperature when gravid to reduce the mismatch with the optimum for embryo development. Specifically, in species with high preferred body temperature, where thermal conflicts are potentially most severe, females significantly reduced their body temperature when gravid (negative values of Hedges’ *g*; Figure 1). Conversely, in species with low preferred body temperatures females increased their body temperature when gravid (positive values of Hedges’ *g*; Figure 1). Combined, this strongly suggests that females with extreme body temperatures regulate their own body temperature to meet the thermal optima of embryos (Supplementary Table S4).

**Figure 2.**
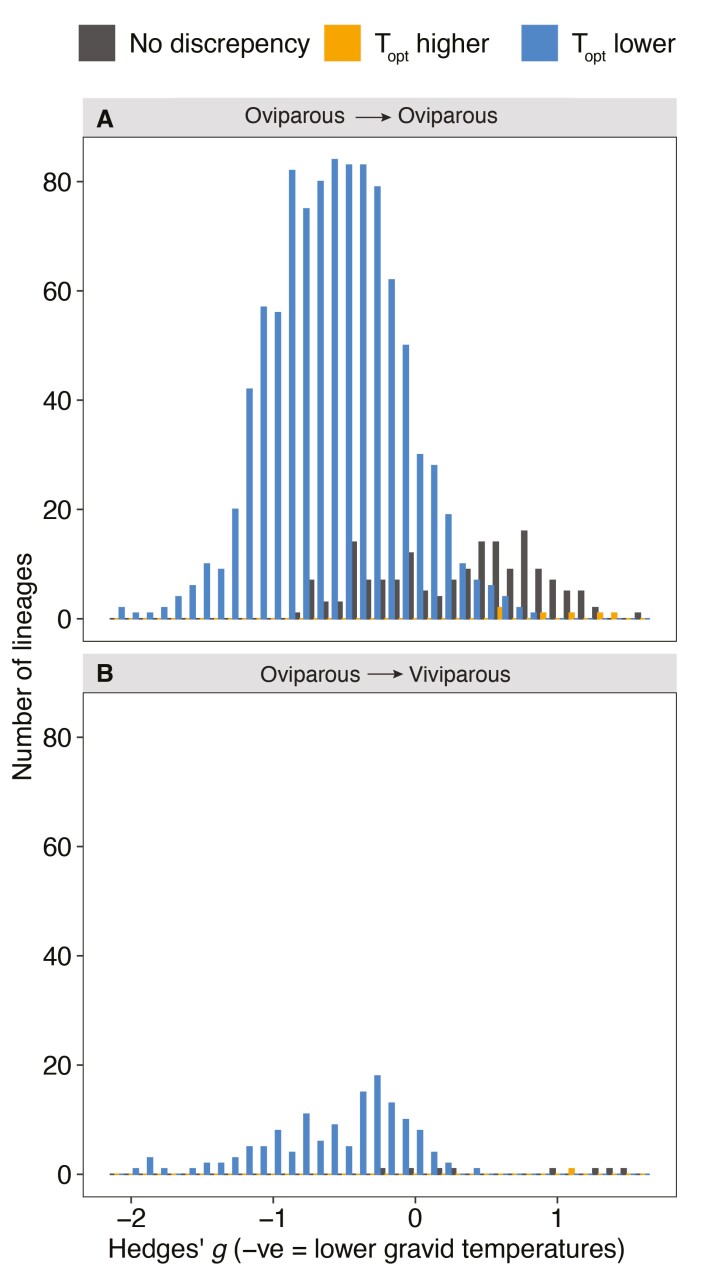
Female behavioral plasticity facilitates transitions to live birth. The adjustment of body temperature by females in lineages where egg laying was maintained (top panel) and where live-bearing evolved (bottom panel) in relation to whether embryos had a significantly lower (*T*_*opt*_ lower; blue), higher (*T*_*opt*_ higher; orange), or aligned (no discrepancy; grey) estimated thermal optimum with adults (see *Methods* section “*Testing the effect of maternal plasticity and the alignment of embryo and adult thermal optima on the evolution of viviparity*” for how mismatches in thermal optima were estimated).

Contrary to the expectation that selection for behavioral plasticity is greater in live-bearing females, the adjustment of body temperature when gravid did not differ between live-bearing and egg-laying females (PM = −0.11, CI: −0.93, 0.61, *pMCMC* = 0.35; Supplementary Table S5). Egg-laying females with higher *P*_*bt*_ downregulated their temperature when gravid, while species with low *P*_*bt*_ upregulated their body temperatures in a similar way to live-bearing females (Figure 2). Phylogenetic correlation between *P*_*bt*_ and Hedges’ *g*: oviparous PM = −0.77, CI: −0.97, −0.05, *pMCMC* = 0.04; viviparous PM = −0.90, CI: −0.98, −0.29, *pMCMC* = 0.01; Supplementary Table S6). Ancestral reconstructions of Hedges’ *g* also showed that, in lineages where there were larger thermal mismatches between adults and embryos, females adjusted their body temperature to a much greater extent than when female and embryo thermal optima were aligned, irrespective of whether the species were egg-laying or live-bearing (Figure 2), MR-BPMM: oviparous ancestors Hedges’ *g* PM = −1.05, CI = −2.54, −0.33, *pMCMC* = 0.001. Viviparous ancestors Hedges’ *g* PM = −1.04, CI = −1.54, −0.44, *pMCMC* = 0.001; Supplementary Table S8). Consequently, estimates of Hedges’ *g* did not differ between the ancestors of oviparous (Figure 2A) and viviparous (Figure 2B) species. This suggests that behavioral plasticity was present prior to the emergence of live birth.

### Does viviparity evolve more readily when adult and embryo thermal optima are well aligned?

**Figure 3.**
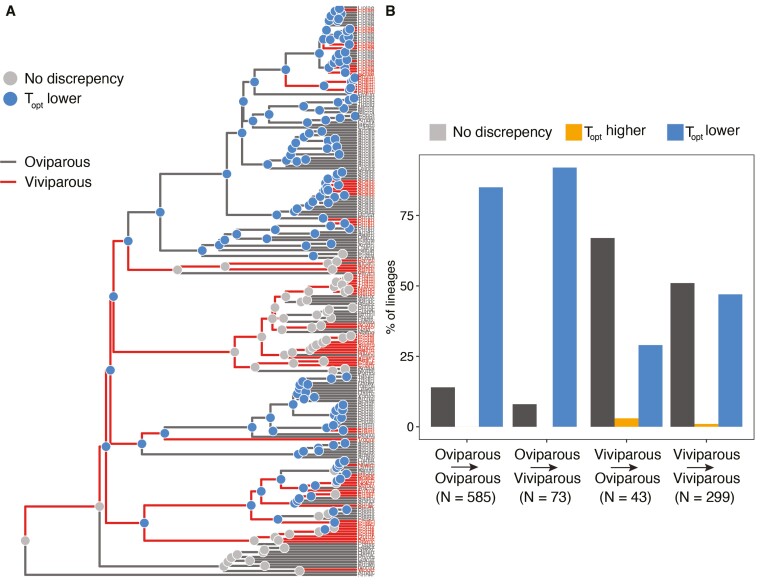
Alignment of embryo and adult thermal optima and transitions to viviparity across 224 species of squamate reptiles. (A) Tip labels and branches are colored according to reproductive modes (red = live bearing/viviparous, grey = egg laying/oviparous; branch colors represent predicted ancestral values; Supplementary Table S7). Colored nodes correspond to the discrepancy between *P*_*bt*_ and *T*_*opt*_ (grey = no discrepancy, blue = *T*_*opt*_ < *P*_*bt*_). (B) Percentage of lineages with transitions between reproductive modes and the discrepancy between *P*_*bt*_ and *T*_*opt*_ (grey = no discrepancy, blue = *T*_*opt*_ < *P*_*bt*_, and orange = *T*_*opt*_ > *P*_*bt*_).

The presence of female thermal plasticity in egg-laying and live-bearing species suggests it may circumvent the barriers to the evolution of live-bearing imposed by mismatches in slowly evolving thermal optima of adults and embryos. However, this does not rule out the alternative hypothesis that viviparity evolves predominantly in lineages where adult and embryo thermal optima are aligned to begin with, alleviating the potential costs to females of adjusting their body temperatures. Estimates of *P*_*bt*_ and *T*_*opt*_ in the egg-laying ancestors of live-bearing species showed that they were no more likely to have aligned adult and embryo thermal optima than the ancestors of egg-laying species. Specifically, 8% of ancestors of live-bearing species had aligned embryo and adult thermal optima compared to 14% in the ancestors of egg-laying species (Figure 3, χ^2^ = 1.5, *df* = 1, *p* > .05; Supplementary Table S9). Consequently, in 92% of the oviparous ancestors of viviparous species, there were mismatches between the predicted thermal optima of embryos and adults, illustrating a widespread need for female plasticity to resolve thermal conflicts (Supplementary Table S9).

## Discussion

Our results suggest that the thermal optima of embryos are commonly lower than the preferred body temperatures of females in oviparous snakes and lizards. Both adult and embryo thermal biology appear to evolve slowly, generating a widespread and evolutionarily persistent mismatch between the thermal optima of mothers and their embryos. Our data suggest non-gravid females have preferred body temperatures that are on average 4°C higher than the temperature that maximizes hatching success. We show that the retention of embryos at such temperatures would result in a substantial decline in hatching success (~50%) while other studies have shown they can also cause significant developmental malformations (reviewed in Noble et al., 2018b). We found little evidence that female-preferred body temperature and offspring thermal optima coevolve, hence such mismatches could hamper transitions to viviparity without females adjusting their body temperatures to meet the thermal requirements of developing embryos.

Our findings support the hypothesis that behavioral thermoregulation by gravid females eliminates this constraint on the evolution of viviparity (Beuchat, 1986). In species where the preferred body temperature of adults is higher than the thermal optimum of embryos (the typical situation), gravid mothers downregulate their body temperature by reducing basking activity and spending more time in cooler microhabitats (Mathies & Andrews, 1997). We also show that the converse is true, that species with low preferred body temperatures can upregulate their body temperature and accelerate the development of their embryos via increased basking and shivering thermogenesis (Shine, 2006; Shine et al., 1997). While we did not test this, mothers may also provide more stable developmental temperatures by retaining embryos closer to their thermal optima (Webb et al., 2006).

Our results suggest this capacity for behavioral plasticity is not something that evolved after, or during, transitions to viviparity. The shifts in body temperature between gravid and non-gravid females were phylogenetically correlated with the discrepancy between adult and embryo thermal optima in both oviparous and viviparous species. Ancestral state reconstructions also suggest that this behavioral plasticity was present prior to the emergence of live birth, negating the need for adult and embryo thermal optima to be aligned for viviparity to evolve. The behavioral regulation of body temperature by gravid egg-laying females may appear surprising considering that oviparous species often lay their eggs within the first third of embryonic development commonly around the time of limb bud formation (Andrews & Mathies, 2000). However, early developmental stages involve gastrulation, neurulation, and organogenesis which are potentially more sensitive to thermal stress than later embryonic stages, which are predominantly associated with growth (Beuchat, 1988; Sanger et al., 2018). The temperature sensitivity of early-stage embryos may therefore generate selection for the resolution of mother-offspring thermal conflicts in both egg-laying and live-bearing species. If true, the innovation of live birth may owe its origin to mechanisms of behavioral temperature regulation put in place long before live birth itself evolved.

Behavioral plasticity has continued to play an important role in thermal adaptation over and above facilitating the evolution of live birth. Behavioral plasticity is frequently maintained in viviparous species that have colonized cool climates. Such behavioral flexibility enables females to upregulate their body temperature to maintain embryos at significantly warmer temperatures than the external environment, contributing to the adaptive value of viviparity (Andrews, 2000; Le Henanff et al., 2013; Shine, 1985; Uller & Olsson, 2010; Warner & Shine, 2007). The ability to cope with a greater range of temperatures has the potential to allow populations to persist and expand into suboptimal environments (Feldman et al., 2015; Muñoz, 2022). Female thermoregulatory behavior, therefore, appears to be a key adaptation that helps resolve thermal mismatches between adults and embryos and facilitate the expansion of reptiles into a variety of environments.

## Supplementary Material

qrad031_suppl_Supplementary_Material

qrad031_suppl_Supplementary_Table_S1_S12

## Data Availability

All data and code are publicly available at: https://osf.io/jt28v/.
