# Supplementary material for: Maternal behavioral thermoregulation facilitated evolutionary transitions from egg laying to live birth
Source: Evol Lett. 2023 Jul 26;7(5):351–60. doi: 10.1093/evlett/qrad031 (PMC10565886; doi:10.1093/evlett/qrad031)
Supplement: qrad031_suppl_Supplementary_Material [file qrad031_suppl_supplementary_material.pdf]

Supplementary Material

Maternal behavioural thermoregulation facilitated evolutionary transitions from egg laying to live birth

Amanda K. Pettersen*, Nathalie Feiner, Daniel W.A. Noble, Geoffrey M. While, Tobias Uller† & Charlie K. Cornwallis†

*Corresponding author. Email: amanda.pettersen@sydney.edu.au.

†Joint senior author.

Figures

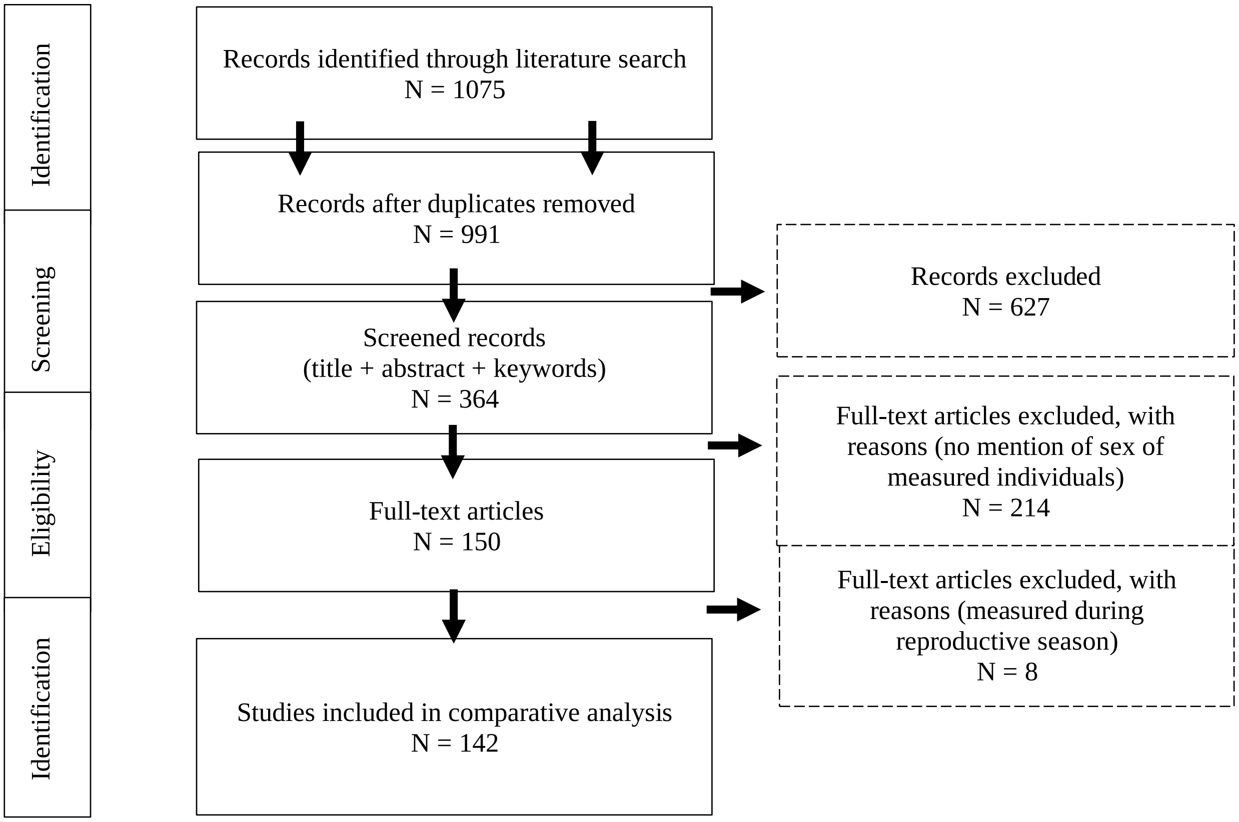

Fig. S1: PRISMA statement for Dataset 2 - Preferred body temperature in non-gravid females (P_{bt}). Our original literature search using ISI *Web of Science* yielded 1075 papers. Of these 84 were either duplicates, or already included in dataset 1 (see above) and were removed, 627 records based on the abstract and 214 full-text articles were rejected based on irrelevance – these studies did not specify sex of the measured individuals. A further 8 records were excluded since individuals were measured during the reproductive season. A resulting 142 articles (supplemented with 42 studies from [10]) were included in the final analysis.

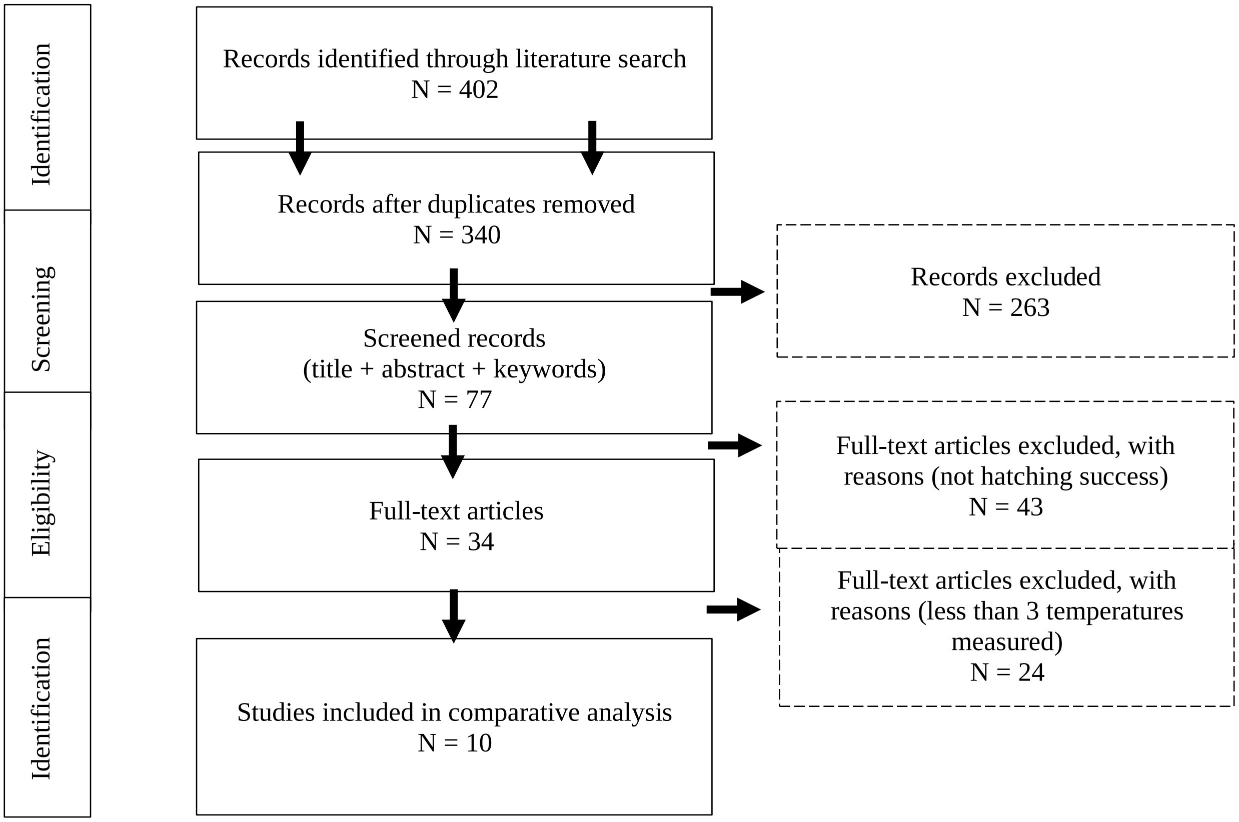

Fig. S2: PRISMA statement for Dataset 3 - Calculation of embryo thermal performance (T_{opt}). Our original literature search using ISI *Web of Science* yielded 402 papers. Of these 62 were duplicates or already included from the Reptile Development Database [4] and were removed, 263 records based on the abstract and 43 full-text articles were rejected based on irrelevance, for example they measured another metric of thermal performance other than hatching success. A further 24 records measured hatching success across less than three temperatures, such that thermal performance curves could not be estimated. A resulting 10 articles were included in the final analysis in addition to those obtained from the Reptile Development Database.

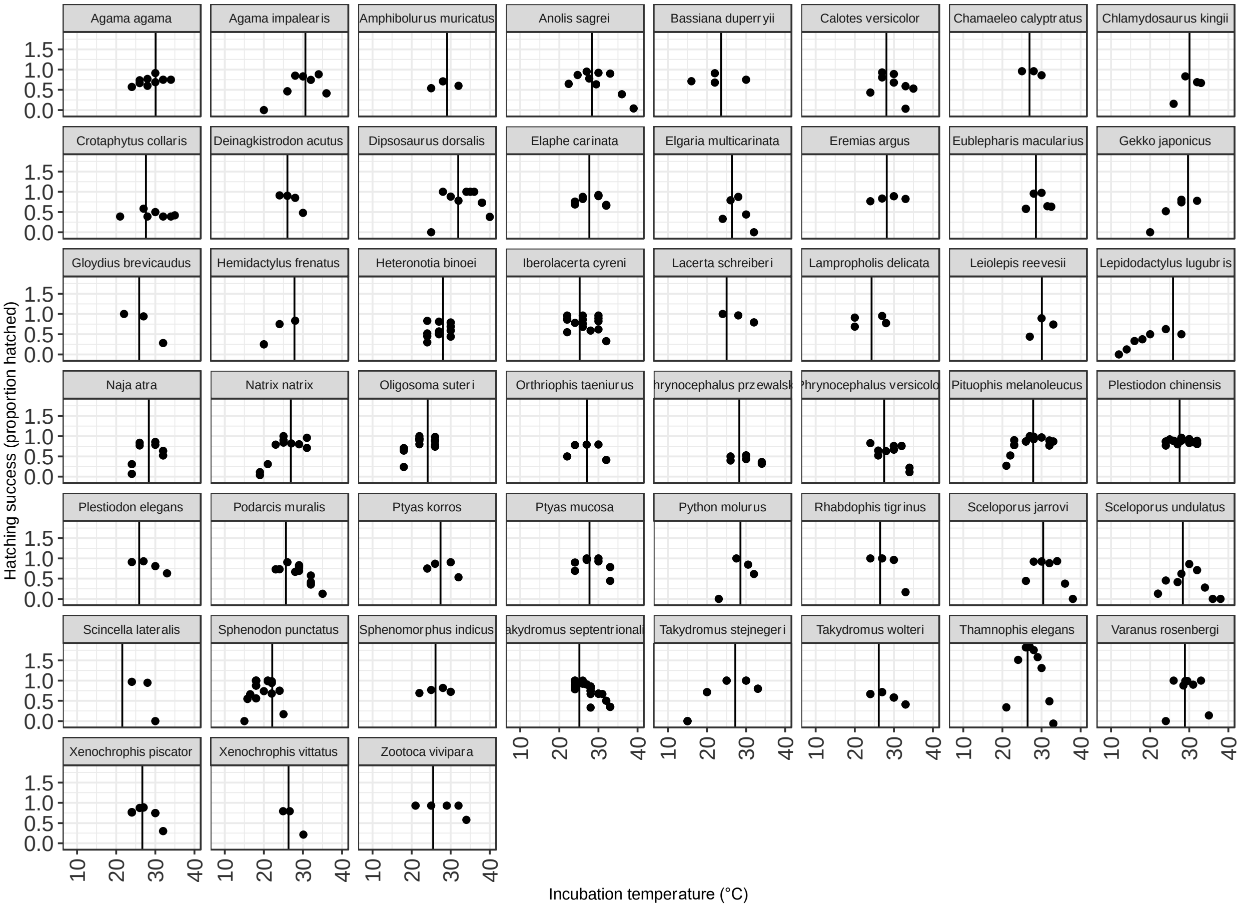

Fig. S3. Raw data used to calculate the temperature that optimizes hatching success (T_{opt}) for 51 oviparous (n = 47) and viviparous (n = 4) species. Plots show species-level hatching success (%) across constant incubation temperatures (°C). Imputed values of T_{opt} and sampling error from a Bayesian Phylogenetic Mixed Effects Model shown by black lines and grey shading, respectively.

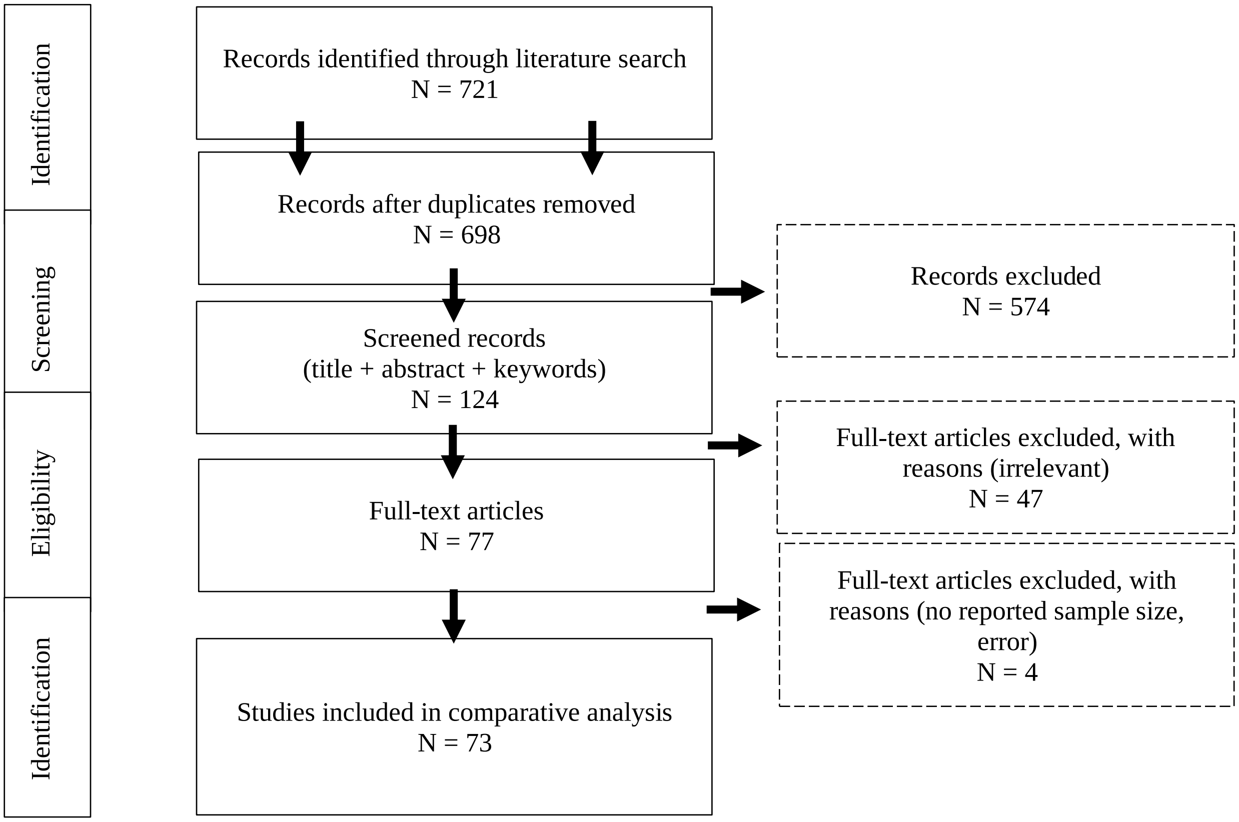

Fig. S4: PRISMA statement for Dataset 1- Preferred body temperature in gravid and non-gravid females and effect size calculation (Hedges' *g*). Our original literature search using ISI *Web of Science* yielded 721 papers. Of these 23 were duplicates and were removed, 574 records based on the abstract and 47 full-text articles were rejected based on irrelevance – these studies did not measure both gravid and non-gravid body temperatures in female squamates. A further 4 records did not report sample size or a statistic from which sample size could be calculated and/or a measure of error from which standard deviation could be determined. A resulting 77 articles representing 54 species were included in the final analysis.

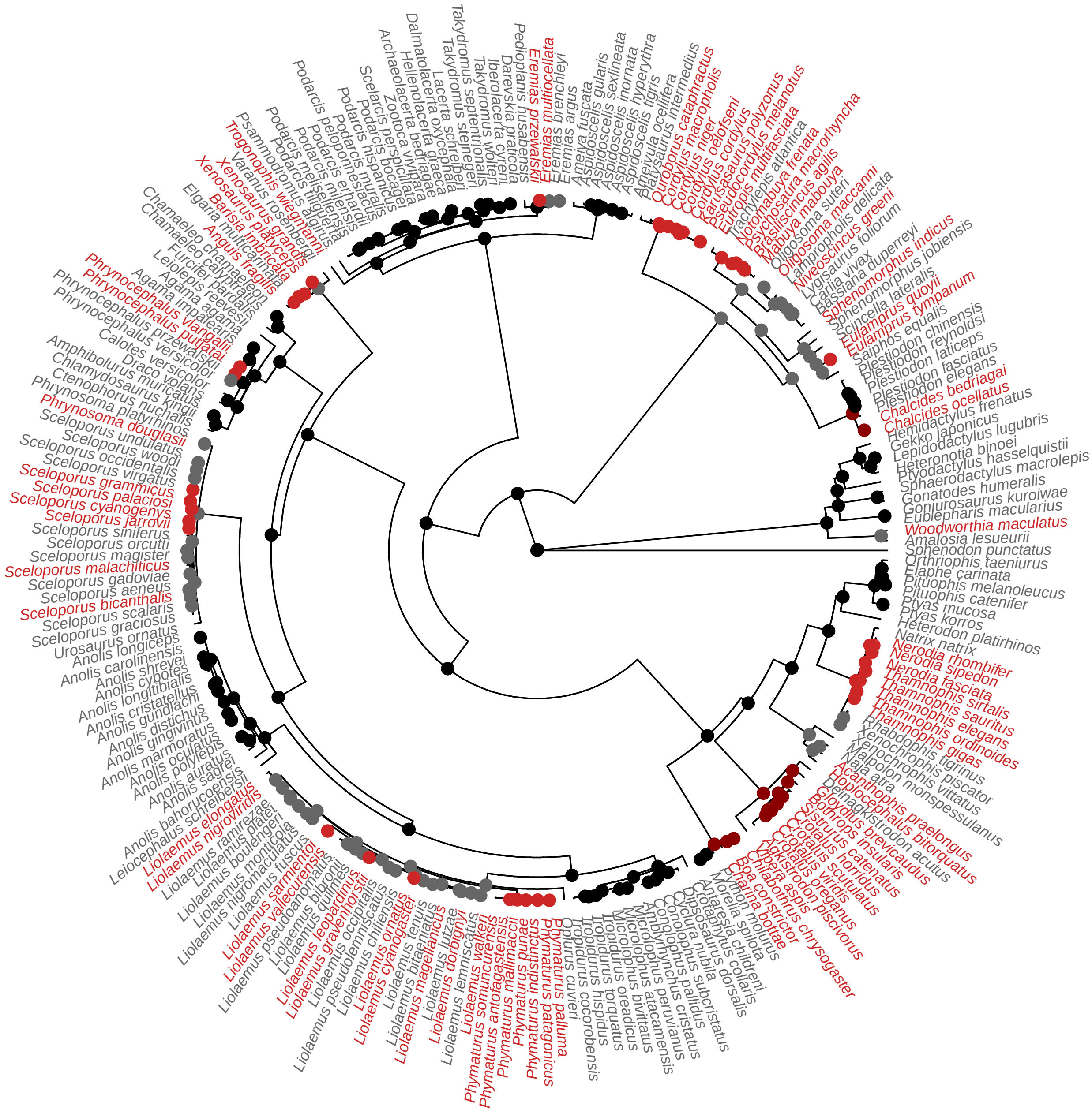

Fig. S5. Changes in the rate of evolution in viviparity across the trimmed phylogeny. Rate shifts were identified using hidden markov models using the R package 'corHMM. Black and dark grey circles indicate oviparous states with different rates of evolution, and dark red and red circles indicate viviparous states with different rates of evolution.

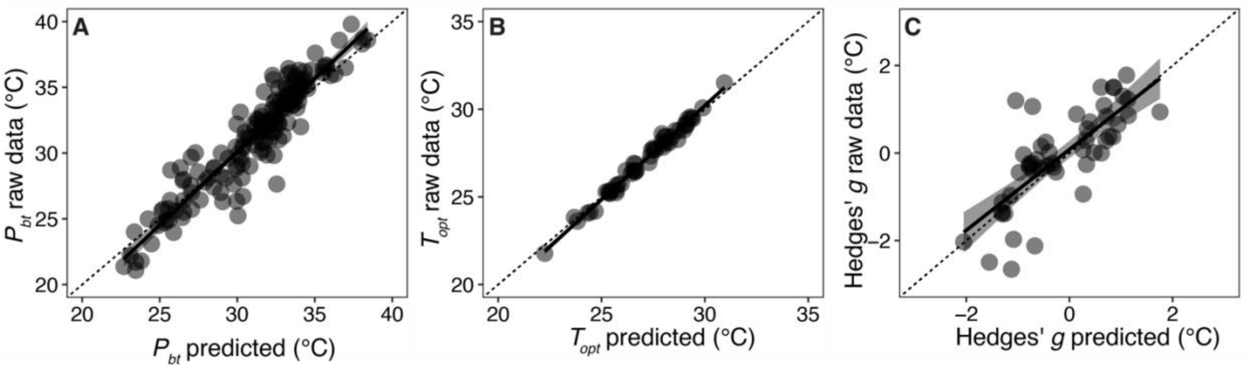

Fig. S6. The relationship between raw data and values predicted by MR-BPMMs. A) preferred body temperature (P_{bt}), B) optimal temperature for embryos (T_{opt}) and C) changes in female body temperature when gravid (Hedges' *g*). Black dotted line represents a 1:1 relationship between raw and predicted values.

References cited in Tables S1 and S12

1. Webb, J. K., Shine, R. & Christian, K. A. The Adaptive Significance of Reptilian Viviparity in the Tropics: Testing the Maternal Manipulation Hypothesis. *Evolution* **60**, 115–122 (2006).
2. Steele, A. L., Wibbels, T. & Warner, D. A. Revisiting the first report of temperature-dependent sex determination in a vertebrate, the African redhead agama. *Journal of Zoology* **306**, 16–22 (2018).
3. Mouden, E. H. E. & Pieau, M. Z. A. C. Effects of incubation temperature on embryonic development and sex determination in the North African agamid lizard, Agama Impalearis. *The Herpetological Journal* **11**, 101–108 (2001).
4. Crane, A. L. & Greene, B. D. The Effect of Reproductive Condition on Thermoregulation in Female Agkistrodon piscivorus Near the Northwestern Range Limit. *herp* **64**, 156–167 (2008).
5. Bartholomew, G. A. & Lasiewski, R. C. Heating and cooling rates, heart rate and simulated diving in the Galapagos marine iguana. *Comparative Biochemistry and Physiology* **16**, 573–582 (1965).
6. Brooks, G. R. Body Temperatures of Three Lizards from Dominica, West Indies. *Herpetologica* **24**, 209–214 (1968).
7. Esquerré, D., Keogh, J. S. & Schwanz, L. E. Direct effects of incubation temperature on morphology, thermoregulatory behaviour and locomotor performance in jacky dragons (Amphibolurus muricatus). *J Therm Biol* **43**, 33–39 (2014).
8. Schwanz, L. E., Hodgson, M. J. & May, A. Costs of thermoregulation in variable thermal environments in the jacky dragon (Amphibolurus muricatus). *Journal of Zoology* **305**, 267–273 (2018).
9. Gregory, P. T. Physical Factor Selectivity in the Fossorial Lizard Anguis fragilis. *Journal of Herpetology* **14**, 95–99 (1980).
10. Meek, R. Null models and the thermal biology of the anguid lizard Anguis fragilis; evidence for thermoregulation? *Amphibia-Reptilia* **26**, 445–450 (2005).
11. Ramírez-Bautista, A. & Benabib, M. Perch Height of the Arboreal Lizard Anolis nebulosus (Sauria: Polychrotidae) from a Tropical Dry Forest of México: Effect of the Reproductive Season. *cope* **2001**, 187–193 (2001).

12. Cast, E. *et al.* Natural history of an anoline lizard community in the Sierra de Baoruco, Dominican Republic. *Caribbean Journal of Science* **36**, 258–266 (2000).

13. Sifers, S. M., Yeska, M. L., Ramos, Y. M., Powell, R. & Parmerlee, J. S. Anolis lizards restricted to altered edge habitats in a Hispaniolan cloud forest. *Caribbean Journal of Science* **37**, 55–62 (2001).

14. Brown, R. P. & Griffin, S. Lower selected body temperatures after food deprivation in the lizard Anolis carolinensis. *Journal of Thermal Biology* **30**, 79–83 (2005).

15. Lailvaux, S. P. & Irschick, D. J. Effects of Temperature and Sex on Jump Performance and Biomechanics in the Lizard Anolis Carolinensis. *Functional Ecology* **21**, 534–543 (2007).

16. Hertz, P. E. Evaluating Thermal Resource Partitioning. By Sympatric Lizards Anolis cooki and A. cristatellus: A Field Test Using Null Hypotheses. *Oecologia* **90**, 127–136 (1992).

17. Hertz, P. E. & Huey, R. B. Compensation for Altitudinal Changes in the Thermal Environment by Some Anolis Lizards on Hispaniola. *Ecology* **62**, 515–521 (1981).

18. Eaton, J. M., Larimer, S. C., Howard, K. G., Powell, R. & Parmerlee, J. S. Population densities and ecological release of the solitary lizard Anolis gingivinus in Anguilla, West Indies. *Caribbean Journal of Science* **38**, 27–36 (2002).

19. Powell, R. Anolis longiceps. *Catalogue of American Amphibians and Reptiles (CAAR)* (1999).

20. Huey, R. B. & Webster, T. P. Thermal Biology of a Solitary Lizard: Anolis Marmoratus of Guadeloupe, Lesser Antilles. *Ecology* **56**, 445–452 (1975).

21. Hertz, P. E. Thermal Passivity of a Tropical Forest Lizard, Anolis polylepis. *Journal of Herpetology* **8**, 323–327 (1974).

22. Sanger, T. J., Kyrkos, J., Lachance, D. J., Czesny, B. & Stroud, J. T. The effects of thermal stress on the early development of the lizard Anolis sagrei. *Journal of Experimental Zoology Part A: Ecological and Integrative Physiology* **329**, 244–251 (2018).

23. Pearson, P. R. & Warner, D. A. Habitat-and season-specific temperatures affect phenotypic development of hatchling lizards. *Biology letters* **12**, 20160646 (2016).

24. Lorioux, S., DeNardo, D. F., Gorelick, R. & Lourdais, O. Maternal influences on early development: preferred temperature prior to oviposition hastens embryogenesis and enhances offspring traits in the Children's python, Antaresia childreni. *J Exp Biol* **215**, 1346–1353 (2012).

25. Lourdais, O., Heulin, B. & Denardo, D. F. Thermoregulation during gravidity in the children's python (Antaresia childreni): a test of the preadaptation hypothesis for maternal thermophily in snakes. *Biological Journal of the Linnean Society* **93**, 499–508 (2008).

26. Bauwens, D., Castilla, A. M. & Mouton, P. le F. N. Field body temperatures, activity levels and opportunities for thermoregulation in an extreme microhabitat specialist, the girdled lizard (Cordylus macropholis). *Journal of Zoology* **249**, 11–18 (1999).

27. Schall, J. J. Thermal Ecology of Five Sympatric Species of Cnemidophorus (Sauria: Teiidae). *Herpetologica* **33**, 261–272 (1977).

28. Bostic, D. L. Thermoregulation and Hibernation of the Lizard, Cnemidophorus Hyperythrus Beldingi (Sauria: Teiidae). *The Southwestern Naturalist* **11**, 275–289 (1966).

29. Bogert, C. M. Thermoregulation in reptiles; a factor in evolution. *Evolution* **3**, 195–211 (1949).

30. Smith, G. R., Ballinger, R. E. & Lemos-Espinal, J. Temperature relationships of the lizard, Barisia imbricata, from México. *Amphibia-Reptilia* **19**, 95–99 (1998).

31. Telemeco, R. S., Radder, R. S., Baird, T. A. & Shine, R. Thermal effects on reptile reproduction: adaptation and phenotypic plasticity in a montane lizard. *Biol J Linn Soc* **100**, 642–655 (2010).

32. Shine, R. & Harlow, P. S. Maternal Manipulation of Offspring Phenotypes via Nest-Site Selection in an Oviparous Lizard. *Ecology* **77**, 1808–1817 (1996).

33. Chiaraviglio, M. The effects of reproductive condition on thermoregulation in the Argentina boa constrictor (Boa constrictor occidentalis) (Boidae). *hmon* **20**, 172–177 (2006).

34. Bovo, R. P. (Universidade E. P. I. de B. D. de Z., Marques, O. A. V. (Instituto B. L. de E. e E. & Andrade, D. V. (Universidade E. P. I. de B. D. de Z. Does gestation or feeding affect the body temperature of the golden lancehead, Bothrops insularis (Squamata: Viperidae) under field conditions? *Zoologia (Curitiba, Impresso)* (2010).

35. Bovo, R. P., Marques, O. A. V. & Andrade, D. V. When Basking Is Not an Option: Thermoregulation of a Viperid Snake Endemic to a Small Island in the South Atlantic of Brazil. *Copeia* **2012**, 408–418 (2012).

36. Shanbhag, B. A., Saidapur, S. K. & Radder, R. S. Lowering body temperature induces embryonic diapause during prolonged egg retention in the lizard, Calotes versicolor. *Naturwissenschaften* **90**, 33–35 (2003).

37. Radder, R. S., Shanbhag, B. A. & Saidapur, S. K. Influence of incubation temperature and substrate on eggs and embryos of the garden lizard, Calotes versicolor (Daud.). *Amphibia-Reptilia* **23**, 71–82 (2002).

38. Ji, X., Qiu, Q.-B. & Diong, C.-H. Influence of incubation temperature on hatching success, energy expenditure for embryonic development, and size and morphology of hatchlings in the oriental garden lizard, Calotes versicolor (Agamidae). *Journal of Experimental Zoology* **292**, 649–659 (2002).

39. Singh, S., Smyth, A. K. & Blomberg, S. P. Thermal ecology and structural habitat use of two sympatric lizards (Carlia vivax and Lygisaurus foliorum) in subtropical Australia. *Austral Ecology* **27**, 616–623 (2002).

40. Hailey, E., Rose, E. A. & Pulford, E. Food consumption, thermoregulation and ecology of the skink Chalcides bedriagai. *The Herpetological Journal* **1**, 144–153 (1987).

41. Daut, E. F. & Andrews, R. M. The Effect of Pregnancy on Thermoregulatory Behavior of the Viviparous Lizard Chalcides ocellatus. *Journal of Herpetology* **27**, 6–13 (1993).

42. Andrews, R. M. Effects of incubation temperature on growth and performance of the veiled chameleon (Chamaeleo calyptratus). *Journal of Experimental Zoology Part A: Ecological Genetics and Physiology* **309A**, 435–446 (2008).

43. Andrews, R. M. Lizards in the slow lane: Thermal biology of chameleons. *Journal of Thermal Biology* **33**, 57–61 (2008).

44. Dorcas, M. E. & Peterson, C. R. Daily Body Temperature Variation in Free-Ranging Rubber Boas. *Herpetologica* **54**, 88–103 (1998).

45. Harlow, P. S. & Shine, R. Temperature-Dependent Sex Determination in the Frillneck Lizard, Chlamydosaurus kingii (Agamidae). *Herpetologica* **55**, 205–212 (1999).

46. Hatano, F. H. *et al.* Thermal ecology and activity patterns of the lizard community of the Restinga of Jurubatiba, Macaé, RJ. *Braz J Biol* **61**, 287–294 (2001).

47. Christian, K., Tracy, C. R. & Porter, W. P. Seasonal Shifts in Body Temperature and Use of Microhabitats by Galapagos Land Iguanas (Conolophus Pallidus). *Ecology* **64**, 463–468 (1983).

48. Snell, H. L. & Christian, K. A. Energetics of Galapagos Land Iguanas: A Comparison of Two Island Populations. *Herpetologica* **41**, 437–442 (1985).

49. Truter, J. C. *et al.* An evaluation of daily, seasonal and population-level variation in the thermal preference of a group-living lizard, Ouroborus cataphractus (Sauria: Cordylidae). *Amphibia-Reptilia* **35**, 391–403 (2014).

50. Clusella-Trullas, S., Terblanche, J. S., van Wyk, J. H. & Spotila, J. R. Low repeatability of preferred body temperature in four species of cordylid lizards: temporal variation and implications for adaptive significance. *Evolutionary Ecology* **21**, 63–79 (2007).

51. McConnachie, S., Alexander, G. J. & Whiting, M. J. Selected Body Temperature and Thermoregulatory Behavior in the Sit-and-Wait Foraging Lizard Pseudocordylus melanotus melanotus. *hmon* **23**, 108–122 (2009).

52. Basson, C. H. & Clusella-Trullas, S. The Behavior-Physiology Nexus: Behavioral and Physiological Compensation Are Relied on to Different Extents between Seasons. *Physiol Biochem Zool* **88**, 384–394 (2015).

53. Gardner-Santana, L. C. & Beaupre, S. J. Timber Rattlesnakes (Crotalus horridus) Exhibit Elevated and Less Variable Body Temperatures during Pregnancy. *Copeia* **2009**, 363–368 (2009).

54. Putman, B. J. & Clark, R. W. Behavioral thermal tolerances of free-ranging rattlesnakes (Crotalus oreganus) during the summer foraging season. *Journal of Thermal Biology* **65**, 8–15 (2017).

55. Cardwell, M. D. The reproductive ecology of Mohave rattlesnakes. *Journal of Zoology* **274**, 65–76 (2008).

56. Charland, M. B. & Gregory, P. T. The Influence of Female Reproductive Status on Thermoregulation in a Viviparous Snake, Crotalus viridis. *Copeia* **1990**, 1089–1098 (1990).

57. Gier, P. J., Wallace, R. L. & Ingerman, R. L. Influence of pregnancy on behavioral thermoregulation in the Northern Pacific rattlesnake Crotalus viridis oreganus. *Journal of Experimental Biology* **145**, 465–469 (1989).

58. Graves, B. M. & Duvall, D. Reproduction, rookery use, and thermoregulation in free-ranging, pregnant Crotalus v. viridis. *Journal of Herpetology* 33–41 (1993).

59. Kissner, K. J. Antipredator behaviour and morphology of western plains garter snakes (Thamnophis radix haydeni) and prairie rattlesnakes (Crotalus viridis viridis). (1997).

60. Uzee, E. M. Effects of Thermal Constraints on the Daily Activity of Crotaphytus collaris. (1990).

61. Santoyo-Brito, E., Anderson, M. & Fox, S. Incubation Temperature Modifies Sex Ratio of Hatchlings in Collared Lizards, Crotaphytus collaris. *hpet* **51**, 197–201 (2017).

62. Heatwole, H. Thermal Ecology of the Desert Dragon Amphibolurus inermis. *Ecological Monographs* **40**, 425–457 (1970).

63. Christian, K. A. *et al.* Thermoregulation and Energetics of a Population of Cuban Iguanas (Cyclura nubila) on Isla Magueyes, Puerto Rico. *Copeia* **1986**, 65–69 (1986).

64. Scheers, H. & Van Damme, R. Micro-scale differences in thermal habitat quality and a possible case of evolutionary flexibility in the thermal physiology of lacertid lizards. *Oecologia* **132**, 323–331 (2002).

65. Ćorović, J. & Crnobrnja-Isailović, J. Aspects of thermal ecology of the meadow lizard (Darevskia praticola). *Amphibia-Reptilia* **39**, 229–238 (2018).

66. Lin, Z.-H., Ji, X., Luo, L.-G. & Ma, X.-M. Incubation temperature affects hatching success, embryonic expenditure of energy and hatchling phenotypes of a prolonged egg-retaining snake, Deinagkistrodon acutus (Viperidae). *Journal of Thermal Biology* **30**, 289–297 (2005).

67. Muth, A. Physiological Ecology of Desert Iguana (Dipsosaurus Dorsalis) Eggs: Temperature and Water Relations. *Ecology* **61**, 1335–1343 (1980).

68. Mori, A. & Hikida, T. Natural history observations of the flying lizard, Draco volans sumatranus (Agamidae, Squamata) from Sarawak, Malaysia. *Raffles Bulletin of Zoology* **41**, 83–94 (1993).

69. Ji, X. & Du, W. G. The effects of thermal and hydric environments on hatching success, embryonic use of energy and hatchling traits in a colubrid snake, Elaphe carinata. *Comp Biochem Physiol A Mol Integr Physiol* **129**, 461–471 (2001).

70. Telemeco, R. S. Sex Determination in Southern Alligator Lizards (Elgaria multicarinata; Anguidae). *herp* **71**, 8–11 (2015).

71. Reynolds, R. G. & Gerber, G. P. Ecology and conservation of the Turks Island Boa (Epicrates chrysogaster chrysogaster: Squamata: Boidae) on Big Ambergris Cay. *Journal of Herpetology* **46**, 578–586 (2012).

72. Qilei, H., Hongxia, L. & Xiang, J. Phenotypic variation in hatchling Mongolian racerunners Eremias argus from eggs incubated at constant versus fluctuating temperatures. *Dong Wu Xue Bao* **52**, 1049–1057 (2006).

73. Xu, X.-F. & Ji, X. Ontogenetic shifts in thermal tolerance, selected body temperature and thermal dependence of food assimilation and locomotor performance in a lacertid lizard, Eremias brenchleyi. *Comp Biochem Physiol A Mol Integr Physiol* **143**, 118–124 (2006).
74. Tang, X. *et al.* Ontogenetic and sexual differences of thermal biology and locomotor performance in a lacertid lizard, Eremias multiocellata. *Zoology* **116**, 331–335 (2013).
75. Yue, F. *et al.* Body temperature and standard metabolic rate of the female viviparous lizard Eremias multiocellata during reproduction. *Canadian Journal of Zoology* (2012) doi:10.1139/z11-116.
76. Li, H., Wang, Z., Mei, W. & Ji, X. Temperature acclimation affects thermal preference and tolerance in three Eremias lizards (Lacertidae). *Current Zoology* **55**, 258–265 (2009).
77. Li, H., Qu, Y.-F., Hu, R.-B. & Ji, X. Evolution of viviparity in cold-climate lizards: testing the maternal manipulation hypothesis. *Evol Ecol* **23**, 777–790 (2009).
78. Viets, B. E., Ewert, M. A., Talent, L. G. & Nelson, C. E. Sex-determining mechanisms in squamate reptiles. *Journal of Experimental Zoology* **270**, 45–56 (1994).
79. Schwarzkopf, L. Evidence of Geographic Variation in Lethal Temperature but Not Activity Temperature of a Lizard. *Journal of Herpetology* **32**, 102–106 (1998).
80. Schwarzkopf, L. & Shine, R. Thermal biology of reproduction in viviparous skinks, Eulamprus tympanum: why do gravid females bask more? *Oecologia* **88**, 562–569 (1991).
81. Ji, X., Lin, C.-X., Lin, L.-H., Qiu, Q.-B. & Du, Y. Evolution of viviparity in warm-climate lizards: an experimental test of the maternal manipulation hypothesis. *Journal of Evolutionary Biology* **20**, 1037–1045 (2007).
82. Hong, L., Luxi, M., Linqiang, S. & Xiang, J. Many-lined Sun Skinks (Mabuya multifasciata) Shift Their Thermal Preferences Downwards When Fasted. *Asian Herpetological Research* **1**, 36–39 (2010).
83. Ferguson, G. W. *et al.* Do Panther Chameleons Bask to Regulate Endogenous Vitamin D3 Production? *Physiological and Biochemical Zoology* **76**, 52–59 (2003).
84. Tokunaga, S. Temperature-Dependent Sex Determination in Gekko japonicus (Gekkonidae, Reptilia) (temperature-dependent sex determination/Gekko japonicus/sex differentiation/Reptilia). *Development, growth & differentiation* **27**, 117–120 (1985).

85. Gao, J.-F., Qu, Y.-F., Luo, L.-G. & Ji, X. Evolution of reptilian viviparity: a test of the maternal manipulation hypothesis in a temperate snake, Gloydius brevicaudus (Viperidae). *Zoological science* **27**, 248–255 (2010).

86. Miranda, J. P., Ricci-Lobão, A. & Rocha, C. F. D. Influence of structural habitat use on the thermal ecology of Gonatodes humeralis (Squamata: Gekkonidae) from a transitional forest in Maranhão, Brazil. *Zoologia (Curitiba)* **27**, 35–39 (2010).

87. Werner, Y. L., Takahashi, H., Yasukawa, Y. & Ota, H. Factors affecting foraging behaviour, as seen in a nocturnal ground lizard, Goniurosaurus kuroiwae kuroiwae. *Journal of Natural History* **40**, 439–459 (2006).

88. Pafilis, P., Maragou, P., Sagonas, K. & Valakos, E. Partitioning thermal habitat on a vertical rock, a herculean task. *Journal of Thermal Biology* **70**, 54–60 (2017).

89. Ota, H. Female reproductive cycles in the northernmost populations of the two gekkonid lizards,Hemidactylus frenatus andLepidodactylus lugubris. *Ecol. Res.* **9**, 121–130 (1994).

90. Plummer, M. V. & Mills, N. E. Body Temperature Variation in Free-Ranging Hognose Snakes (Heterodon platirhinos). *hpet* **44**, 471–474 (2010).

91. Kearney, M. & Shine, R. Developmental success, stability, and plasticity in closely related parthenogenetic and sexual lizards (Heteronotia, Gekkonidae). *evol* **58**, 1560–1572 (2004).

92. Fitzgerald, M., Shine, R. & Lemckert, F. A reluctant heliotherm: thermal ecology of the arboreal snake Hoplocephalus stephensii (Elapidae) in dense forest. *Journal of Thermal Biology* **28**, 515–524 (2003).

93. Rock, J., Andrews, R. M. & Cree, A. Effects of reproductive condition, season, and site on selected temperatures of a viviparous gecko. *Physiol Biochem Zool* **73**, 344–355 (2000).

94. Rock, J., Cree, A. & Andrews, R. M. The effect of reproductive condition on thermoregulation in a viviparous gecko from a cool climate. *Journal of Thermal Biology* **27**, 17–27 (2002).

95. Werner, Y. L. & Whitaker, A. H. Observations and comments on the body temperatures of some New Zealand reptiles. *null* **5**, 375–393 (1978).

96. Rock, J. & Cree, A. Extreme variation in body temperature in a nocturnal thigmothermic lizard. *The Herpetological Journal* **18**, 69–76 (2008).

97. Monasterio, C., Salvador, A., Iraeta, P. & Díaz, J. A. The effects of thermal biology and refuge availability on the restricted distribution of an alpine lizard. *Journal of Biogeography* **36**, 1673–1684 (2009).

98. García-Roa, R., Ortega, J., López Martínez, P. & Martín Rueda, J. Coloración atípica en una hembra de Iberolacerta cyreni en la Sierra de Guadarrama (Madrid). *Asociación Herpetológica Española* (2015) doi:10.13039/501100004837.

99. Monasterio, C., Verdu-Ricoy, J., Salvador, A. & Diaz, J. A. Living at the edge: lower success of eggs and hatchlings at lower elevation may shape range limits in an alpine lizard. *Biological Journal of the Linnean Society* **118**, 829–841 (2016).

100. Monasterio, C., Shoo, L. P., Salvador, A., Siliceo, I. & Diaz, J. A. Thermal constraints on embryonic development as a proximate cause for elevational range limits in two Mediterranean lacertid lizards. *Ecography* **34**, 1030–1039 (2011).

101. Monasterio, C., Shoo, L. P., Salvador, A., Iraeta, P. & Díaz, J. A. High temperature constrains reproductive success in a temperate lizard: implications for distribution range limits and the impacts of climate change. *Journal of Zoology* **291**, 136–145 (2013).

102. Downes, S. J. & Shine, R. Do incubation-induced changes in a lizard's phenotype influence its vulnerability to predators? *Oecologia* **120**, 9–18 (1999).

103. Bilcke, J., Downes, S. & Büscher, I. Combined effect of incubation and ambient temperature on the feeding performance of a small ectotherm. *Austral Ecology* **31**, 937–947 (2006).

104. Marcellini, D. L. & Jenssen, T. A. Thermal Ecology of the Tropical Iguanid Lizard, Leiocephalus schreibersi. *The American Midland Naturalist* **122**, 44–50 (1989).

105. Chixian, L., Yu, D., Qingbo, Q. & Xiang, J. Relatively high but narrow incubation temperatures in lizards depositing eggs in warm and thermally stable nests. 437–445 (2010).

106. Labra, A., Pienaar, J. & Hansen, T. F. Evolution of thermal physiology in Liolaemus lizards: adaptation, phylogenetic inertia, and niche tracking. *Am Nat* **174**, 204–220 (2009).

107. Medina, M., Gutierrez, J., Scolaro, A. & Ibargüengoytía, N. Thermal responses to environmental constraints in two populations of the oviparous lizard Liolaemus bibronii in Patagonia, Argentina. *Journal of Thermal Biology* **34**, 32–40 (2009).

108. Medina, M., Scolaro, A., Méndez-De la Cruz, F., Sinervo, B. & Ibargüengoytía, N. Thermal relationships between body temperature and environment conditions set upper distributional limits on oviparous species. *Journal of Thermal Biology* **36**, 527–534 (2011).
109. Ibargüengoytía, N. R. & Cussac, V. E. Body temperatures of two viviparous Liolaemus lizard species, in Patagonian rain forest and steppe. *The Herpetological Journal* **12**, 131–134 (2002).
110. Labra, A. & Bozinovic, F. Interplay between pregnancy and physiological thermorégulation in Liolaemus lizards. *Écoscience* **9**, 421–426 (2002).
111. Carothers, J. H., Marquet, P. A. & Jaksic, F. M. Thermal ecology of a Liolaemus lizard assemblage along an Andean altitudinal gradient in Chile. *Revista Chilena de Historia Natural* **71**, 39–50 (1998).
112. Almeida-Santos, P., Militão, C. M., Nogueira-Costa, P., Menezes, V. A. & Rocha, C. F. D. Thermal ecology of five remaining populations of an endangered lizard (Liolaemus lutzae) in different restinga habitats in Brazil. *Journal of coastal conservation* (2015).
113. Maia-Carneiro, T. & Rocha, C. F. D. Seasonal variations in behaviour of thermoregulation in juveniles and adults Liolaemus lutzae (Squamata, Liolaemidae) in a remnant of Brazilian restinga. *Behavioural processes* **100**, 48–53 (2013).
114. Jaksić, F. M. & Schwenk, K. Natural History Observations on Liolaemus magellanicus, the Southernmost Lizard in the World. *Herpetologica* **39**, 457–461 (1983).
115. Bujes, C. S. & Verrastro, L. Thermal biology of Liolaemus occipitalis (Squamata, Tropiduridae) in the coastal sand dunes of Rio Grande do Sul, Brazil. *Brazilian Journal of Biology* **66**, 945–954 (2006).
116. Martins, L. S., Verrastro, L. & Tozetti, A. M. The Influences of Habitat on Body Temperature Control in a Southern Population of Liolaemus Occipitalis (Boulenger, 1885) in Brazil. *sajh* **9**, 9–13 (2014).
117. Marquet, P. A., Ortíz, J. C., Bozinovié, F. & Jaksié, F. M. Ecological aspects of thermoregulation at high altitudes: the case of andean Liolaemus lizards in northern Chile. *Oecologia* **81**, 16–20 (1989).
118. Villavicencio, H., Acosta, J., Cánovas, M. & Marinero, J. Thermal ecology of a population of the lizard, Liolaemus pseudoanomalus in western Argentina. *Amphibia-Reptilia* **28**, 163–165 (2007).

119. Robles, C. I. & Halloy, M. Thermal ecology of two syntopic lizard species of the genus Liolaemus. *North-Western Journal of Zoology* (2016).

120. Fernández, J. B., Kubisch, E. L. & Ibargüengoytía, N. R. Viviparity advantages in the lizard Liolaemus sarmientoi from the end of the world. *Evolutionary Biology* **44**, 325–338 (2017).

121. Vrcibradic, D. & Rocha, C. F. D. Field Body Temperatures of Pregnant and Nonpregnant Females of Three Species of Viviparous Skinks (Mabuya) from Southeastern Brazil. *hpet* **38**, 447–451 (2004).

122. Rocha, C. F. D. & Vrcibradic, D. Thermal ecology of two sympatric skinks (Mabuya macrorhyncha and Mabuya agilis) in a Brazilian restinga habitat. *Australian Journal of Ecology* **21**, 110–113 (1996).

123. Blázquez, M. C. Body Temperature, Activity Patterns and Movements by Gravid and Non-Gravid Females of Malpolon monspessulanus. *Journal of Herpetology* **29**, 264–266 (1995).

124. Sepúlveda, M., Vidal, M. A., Fariña, J. M. & Sabat, P. Seasonal and geographic variation in thermal biology of the lizard Microlophus atacamensis (Squamata: Tropiduridae). *Journal of Thermal Biology* **33**, 141–148 (2008).

125. Arguedas, R. *et al.* Haematology and biochemistry of the San Cristóbal Lava Lizard (Microlophus bivittatus). *Conservation physiology* **6**, coy046 (2018).

126. Catenazzi, A., Carrillo, J. & Donnelly, M. A. Seasonal and geographic eurythermy in a coastal Peruvian lizard. *Copeia* **2005**, 713–723 (2005).

127. Huey, R. B. Winter thermal ecology of the iguanid lizard Tropidurus peruvianus. *Copeia* 149–155 (1974).

128. Bryant, G. L., Tores, P. J. D., Warren, K. A. & Fleming, P. A. Does body size influence thermal biology and diet of a python (Morelia spilota imbricata)? *Austral Ecology* **37**, 583–591 (2012).

129. Slip, D. J. & Shine, R. Thermoregulation of Free-Ranging Diamond Pythons, Morelia spilota (Serpentes, Boidae). *Copeia* **1988**, 984–995 (1988).

130. Ji, X. & Du, W.-G. Effects of Thermal and Hydric Environments on Incubating Eggs and Hatchling Traits in the Cobra, Naja naja atra. *Journal of Herpetology* **35**, 186–194 (2001).

131. Shine, R. Reptilian viviparity in cold climates: testing the assumptions of an evolutionary hypothesis. *Oecologia* **57**, 397–405 (1983).

132. Löwenborg, K., Shine, R., Kärvemo, S. & Hagman, M. Grass snakes exploit anthropogenic heat sources to overcome distributional limits imposed by oviparity. *Functional Ecology* **24**, 1095–1102 (2010).

133. Löwenborg, K., Gotthard, K. & Hagman, M. How a thermal dichotomy in nesting environments influences offspring of the world's most northerly oviparous snake, Natrix natrix (Colubridae). *Biological Journal of the Linnean Society* **107**, 833–844 (2012).

134. Isaac, L. A. & Gregory, P. T. Thermoregulatory behaviour of gravid and non-gravid female grass snakes (Natrix natrix) in a thermally limiting high-latitude environment. *Journal of Zoology* **264**, 403–409 (2004).

135. Osgood, D. W. Thermoregulation in Water Snakes Studied by Telemetry. *Copeia* **1970**, 568–571 (1970).

136. Tu, M.-C. & Hutchison, V. H. Influence of pregnancy on thermoregulation of water snakes (Nerodia rhombifera). *Journal of Thermal Biology* **19**, 255–259 (1994).

137. Brown, G. P. & Weatherhead, P. J. Thermal Ecology and Sexual Size Dimorphism in Northern Water Snakes, Nerodia Sipedon. *Ecological Monographs* **70**, 311–330 (2000).

138. Kabat, A. P. Maternal costs of reproduction in the southern snow skink, Niveoscincus microlepidotus. (University of Tasmania, 1999).

139. Schlesinger, C. A. & Shine, R. Selection of Diurnal Retreat Sites by the Nocturnal Gekkonid Lizard Oedura Lesueurii. *Herpetologica* **50**, 156–163 (1994).

140. Dayananda, B., Ibargüengoytía, N., Whiting, M. J. & Webb, J. K. Effects of pregnancy on body temperature and locomotor performance of velvet geckos. *J Therm Biol* **65**, 64–68 (2017).

141. Hare, J. R., Holmes, K. M., Wilson, J. L. & Cree, A. Modelling exposure to selected temperature during pregnancy: the limitations of squamate viviparity in a cool-climate environment. *Biological Journal of the Linnean Society* **96**, 541–552 (2009).

142. Hare, K. M., Daugherty, C. H. & Cree, A. Incubation regime affects juvenile morphology and hatching success, but not sex, of the oviparous lizard Oligosoma suteri (Lacertilia: Scincidae). *New Zealand Journal of Zoology* **29**, 221–229 (2002).

143. Hare, K. M., Longson, C. G., Pledger, S. & Daugherty, C. H. Size, Growth, and Survival Are Reduced at Cool Incubation Temperatures in the Temperate Lizard Oligosoma suteri (Lacertilia: Scincidae). *cope* **2004**, 383–390 (2004).

144. Chapple, D. G., Keall, S. N., Daugherty, C. H. & Hare, K. M. Nest-site selection and the factors influencing hatching success and offspring phenotype in a nocturnal skink. *Amphibia-Reptilia* **38**, 363–369 (2017).

145. Randriamahazo, H. J. a. R. & Mori, A. Thermal Biology of an Iguanian Lizard, *Oplurus cuvieri cuvieri*, in a Tropical Dry Forest of Madagascar. *Current herpetology* **23**, 53–62 (2004).

146. Du, W.-G. & Ji, X. The Effects of Incubation Temperature On Hatching Success, Embryonic Use of Energy and Hatchling Morphology in the Stripe-tailed Ratsnake Elaphe taeniura. *Asiatic Herpetological Research* **11**, 24–30 (2008).

147. Kirchhof, S. *et al.* Thermoregulatory behavior and high thermal preference buffer impact of climate change in a Namib Desert lizard. *Ecosphere* **8**, e02033 (2017).

148. Tang, X. *et al.* Effects of Thermal and Hydric Conditions on Egg Incubation and Hatchling Phenotypes in Two Phrynocephalus Lizards. *Asian Herpetological Research* **3**, 184–191 (2012).

149. Wang, Z., Ma, L., Shao, M. & Ji, X. Are viviparous lizards more vulnerable to climate warming because they have evolved reduced body temperature and heat tolerance? *Oecologia* **185**, 573–582 (2017).

150. Qu, Y., Li, H., Gao, J., Xu, X. & Ji, X. Thermal preference, thermal tolerance and the thermal dependence of digestive performance in two Phrynocephalus lizards (Agamidae), with a review of species studied. *Current Zoology* **57**, 684–700 (2011).

151. Wang, Z., Lu, H.-L., Ma, L. & Ji, X. Viviparity in high-altitude Phrynocephalus lizards is adaptive because embryos cannot fully develop without maternal thermoregulation. *Oecologia* **174**, 639–649 (2014).

152. Christian, K. A. Thermoregulation by the short-horned lizard (Phrynosoma douglassi) at high elevation. *Journal of Thermal Biology* **23**, 395–399 (1998).

153. Powell, G. L. & Russell, A. P. Field thermal ecology of the eastern short-horned lizard (Phrynosoma douglassi brevirostre) in southern Alberta. *Canadian Journal of Zoology* **63**, 228–238 (1985).

154. Lara-Resendiz, R. A., Jezkova, T., Rosen, P. C. & Méndez-de La Cruz, F. R. Thermoregulation during the summer season in the Goode's horned lizard Phrynosoma goodei (Iguania: Phrynosomatidae) in Sonoran Desert. *Amphibia-Reptilia* **35**, 161–172 (2014).

155. Corbalán, V., Debandi, G. & Kubisch, E. Thermal ecology of two sympatric saxicolous lizards of the genus Phymaturus from the Payunia region (Argentina). *Journal of Thermal Biology* **38**, 384–389 (2013).
156. Gómez Alés, R., Acosta, J. C. & Laspiur, A. Thermal biology in two syntopic lizards, Phymaturus extrilidus and Liolaemus parvus, in the Puna region of Argentina. *Journal of Thermal Biology* **68**, 73–82 (2017).
157. Vicenzi, N., Corbalán, V., Miles, D., Sinervo, B. & Ibargüengoytía, N. Range increment or range detriment? Predicting potential changes in distribution caused by climate change for the endemic high-Andean lizard Phymaturus palluma. *Biological Conservation* **206**, 151–160 (2017).
158. Duran, F., Kubisch, E. L. & Boretto, J. M. Thermal physiology of three sympatric and syntopic Liolaemidae lizards in cold and arid environments of Patagonia (Argentina). *J Comp Physiol B* **188**, 141–152 (2018).
159. Ibargüengoytía, N. R. *et al.* Field thermal biology in Phymaturus lizards: Comparisons from the Andes to the Patagonian steppe in Argentina. *Journal of Arid Environments* **72**, 1620–1630 (2008).
160. Ibargüengoytía, N. R. Field, selected body temperature and thermal tolerance of the syntopic lizards Phymaturus patagonicus and Liolaemus elongatus (Iguania: Liolaemidae). *Journal of Arid Environments* **62**, 435–448 (2005).
161. Himes, J. G., Hardy, L. M., Rudolph, D. C. & Burgdorf, S. J. Body temperature variations of the Louisiana pine snake (*Pituophis ruthveni*) in a longleaf pine ecosystem. *Herpetological Natural History. 9(2): 117-126* (2006).
162. Kapfer, J. M., Pauers, M. J., Reineke, D. M., Coggins, J. R. & Hay, R. Environmental, behavioral, and habitat variables influencing body temperature in radio-tagged bullsnakes, Pituophis catenifer sayi. *Journal of Thermal Biology* **33**, 174–179 (2008).
163. Burger, J., Zappalorti, R. T. & Gochfeld, M. Developmental effects of incubation temperature on hatchling pine snakes Pituophis melanoleucus. *Comparative Biochemistry and Physiology Part A: Physiology* **87**, 727–732 (1987).
164. Burger, J. Antipredator behaviour of hatchling snakes: effects of incubation temperature and simulated predators. *Animal Behaviour* **56**, 547–553 (1998).

165. Shen, W., Pei, J. C., Lin, L. H. & Ji, X. Effects of constant versus fluctuating incubation temperatures on hatching success, incubation length and hatchling morphology in the Chinese skink (Plestiodon chinensis). *Asian Herpetol Res* **8**, 262–268 (2017).

166. Ma, L., Buckley, L. B., Huey, R. B. & Du, W.-G. A global test of the cold-climate hypothesis for the evolution of viviparity of squamate reptiles. *Global Ecology and Biogeography* **27**, 679–689 (2018).

167. Du, W.-G., Shou, L., Shen, J.-Y. & Lu, Y.-W. Influence of fluctuating incubation temperatures on hatchling traits in a Chinese skink, Eumeces chinensis. *The Herpetological Journal* **15**, 139–142 (2005).

168. Du, W. G., Shou, L. & Shen, J. Y. Habitat selection in two sympatric Chinese skinks, Eumeces elegans and Sphenomorphus indicus: do thermal preferences matter? *Canadian journal of zoology* **84**, 1300–1306 (2006).

169. Du, W.-G., Yan, S.-J. & Ji, X. Selected body temperature, thermal tolerance and thermal dependence of food assimilation and locomotor performance in adult blue-tailed skinks, Eumeces elegans. *Journal of Thermal Biology* **25**, 197–202 (2000).

170. Shou, L., Du, W.-G. & Liu, J.-K. The effect of incubation temperature on egg survival, hatchling traits and embryonic use of energy in the blue-tailed skink, Eumeces elegans. *Animal Biology* **53**, 27–36 (2003).

171. Brattstrom, B. H. Body Temperatures of Reptiles. *The American Midland Naturalist* **73**, 376–422 (1965).

172. Pentecost, E. D. Behavior of Eumeces laticeps Exposed to a Thermal Gradient. *Journal of Herpetology* **8**, 169–173 (1974).

173. Andrews, R. M. Activity and Thermal Biology of the Sand-Swimming Skink Neoseps reynoldsi: Diel and Seasonal Patterns. *Copeia* **1994**, 91–99 (1994).

174. Carneiro, D., García-Muñoz, E., Kalionzopoulou, A., Llorente, G. A. & Carretero, M. A. Comparing ecophysiological traits in two Podarcis Wall lizards with overlapping ranges. *Salamandra* **51**, 335–344 (2015).

175. Pafilis, P., Lymberakis, P., Sagonas, K. & Valakos, E. The particularities of a remote islet shape the thermoregulatory profile of an endemic Mediterranean lizard. *J Therm Biol* **61**, 55–60 (2016).

176. Castilla, A. M. & Bauwens, D. Thermal biology, microhabitat selection, and conservation of the insular lizard Podarcis hispanica atrata. *Oecologia* **85**, 366–374 (1991).
177. Gabirot, M., Balleri, A., López, P. & José, M. Differences in Thermal Biology Between Two Morphologically Distinct Populations of Iberian Wall Lizards Inhabiting Different Environments. *anzf* **50**, 225–236 (2013).
178. Adamopoulou, C. & Valakos, E. D. Thermal ecology and activity cycle of podarcis milensis in a sandy coastal area. *null* **51**, 39–52 (2005).
179. Braña, F. Summer activity patterns and thermoregulation in the wall l izard, Podarcis muralis. *The Herpetological Journal* **1**, 544–549 (1991).
180. Le Henanff, M., Meylan, S. & Lourdais, O. The sooner the better: reproductive phenology drives ontogenetic trajectories in a temperate squamate (Podarcis muralis). *Biol J Linn Soc* **108**, 384–395 (2013).
181. Van Damme, R., Bauwens, D., Braña, F. & Verheyen, R. F. Incubation Temperature Differentially Affects Hatching Time, Egg Survival, and Hatchling Performance in the Lizard Podarcis muralis. *Herpetologica* **48**, 220–228 (1992).
182. Ji, X. & Braña, F. The influence of thermal and hydric environments on embryonic use of energy and nutrients, and hatchling traits, in the wall lizards (Podarcis muralis). *Comparative Biochemistry and Physiology Part A: Molecular & Integrative Physiology* **124**, 205–213 (1999).
183. Braña, F. Shifts in Body Temperature and Escape Behaviour of Female Podarcis muralis during Pregnancy. *Oikos* **66**, 216–222 (1993).
184. Tosini, G. & Avery, R. Pregnancy decreases set point temperatures for behavioural thermoregulation in the wall lizard Podarcis muralis. *The Herpetological Journal* **6**, 94–96 (1996).
185. Van Damme, R., Bauwens, D., Castilla, A. M. & Verheyen, R. F. Altitudinal variation of the thermal biology and running performance in the lizard Podarcis tiliguerta. *Oecologia* **80**, 516–524 (1989).
186. Díaz, J., Monasterio, C., Iraeta, P. & Salvador, A. Effects of gravidity on the locomotor performance and escape behaviour of two lizard populations: the importance of habitat structure. *Behaviour* **147**, 133–150 (2010).

187. Díaz, J. A., Iraeta, P. & Monasterio, C. Seasonality provokes a shift of thermal preferences in a temperate lizard, but altitude does not. *Journal of Thermal Biology* **31**, 237–242 (2006).
188. Iraeta, P., Salvador, A., Monasterio, C. & Díaz, J. A. Effects of gravidity on the locomotor performance and escape behaviour of two lizard populations: the importance of habitat structure. *Behaviour* **147**, 133–150 (2010).
189. Du, W.-G. & Xiang, J. Effects of Incubation Temperature on Duration of Incubation, Hatching Success, and Hatchling traits in The Gray Rat Snake Ptyas Korros (Colubridae). *Acta Ecologica Sinica* **22**, 548–553 (2002).
190. Lin, Z.-H. & Ji, X. Reproductive output and effects of incubation thermal environments on hatchling phenotypes of mucous rat snakes Ptyas mucosus. *Acta Zoologica S inica* **50**, 541–550 (2004).
191. Lin, L.-H., Ma, X.-M., Li, H. & Ji, X. Phenotypic variation in hatchling Chinese ratsnakes (Zaocys dhumnades) from eggs incubated at constant temperatures. *Journal of Thermal Biology* **35**, 28–33 (2010).
192. Vinegar, A. The effects of temperature on the growth and development of embryos of the Indian python, Python molurus (Reptilia: Serpentes: Boidae). *Copeia* **1973**, 171–173 (1973).
193. Li, C. H. & Xiang, J. I. The Effects of Thermal Environments on Duration of Incubation, Hatching Success and Hatchlings Traits in a Colubrid Snake, Rhabdophis tigrinus lateralis (Boie)[J]. *Acta Ecologica Sinica* **11**, (2002).
194. Wu, Q., Parker, S. L. & Thompson, M. B. Selected body temperature, metabolic rate and activity pattern of the Australian fossorial skink, Saiphos equalis. *The Herpetological Journal* **19**, 127–133 (2009).
195. Ortega, Z., Mencía, A. & Pérez-Mellado, V. Sexual differences in behavioral thermoregulation of the lizard Scelarcis perspicillata. *J Therm Biol* **61**, 44–49 (2016).
196. Andrews, R., Cruz, F. M. L., Cruz, M. & Rodríguez-Romero, F. Field and Selected Body Temperatures of the Lizards Sceloporus aeneus and Sceloporus bicanthalis. *Journal of Herpetology* **33**, (1999).
197. Garrick, L. D. Reproductive influences on behavioral thermoregulation in the lizard Sceloporus cyanogenys. *Physiol Behav* **12**, 85–91 (1974).

198. Woolrich-Piña, G. A. *et al.* Thermal ecology of the lizard Sceloporus gadoviae (Squamata: Phrynosomatidae) in a semiarid region of southern Puebla, Mexico. *Phyllomedusa: Journal of Herpetology* **11**, 21–27 (2012).

199. Lemos-Espinal, J. A., Smith, G. R. & Ballinger, R. E. Thermal ecology of the lizard,Sceloporus gadoviae, in an arid tropical scrub forest. *Journal of Arid Environments* **35**, 311–319 (1997).

200. Adolph, S. C. Influence of Behavioral Thermoregulation on Microhabitat Use by Two Sceloporus Lizards. *Ecology* **71**, 315–327 (1990).

201. Andrews, R. M., Cruz, F. R. M. de la & Cruz, M. V. S. Body Temperatures of Female Sceloporus grammicus: Thermal Stress or Impaired Mobility? *Copeia* **1997**, 108–115 (1997).

202. Mathies, T. & Andrews, R. M. Influence of pregnancy on the thermal biology of the lizard, Sceloporus jarrovi: why do pregnant females exhibit low body temperatures? *Functional Ecology* **11**, 498–507 (1997).

203. Beuchat, C. A. Reproductive Influences on the Thermoregulatory Behavior of a Live-Bearing Lizard. *Copeia* **1986**, 971–979 (1986).

204. Smith, G. R. & Ballinger, R. E. Temperature relationships in the high-altitude viviparous lizard, Sceloporus jarrovi. *American Midland Naturalist* 181–189 (1994).

205. Gadsden, H. & Estrada-RodrÍguez, J. L. Ecology of the spiny lizard Sceloporus jarrovii in the central Chihuahuan Desert. *The Southwestern Naturalist* **52**, 600–608 (2007).

206. Beuchat, C. A. Temperature effects during gestation in a viviparous lizard. *Journal of Thermal Biology* **13**, 135–142 (1988).

207. Vial, J. L. Comparative field responses to diel and annual thermal regimens among Sceloporine lizards, with specific reference to *Sceloporus malachiticus*. *1* **32**, 1–9 (1984).

208. Mayhew, W. W. Temperature Preferences of Sceloporus orcutti. *Herpetologica* **18**, 217–233 (1963).

209. Güizado-Rodríguez, A., García-Vázquez, U. O. & Solano-Zavaleta, I. THERMOREGULATION BY A POPULATION OF SCELOPORUS PALACIOSI FROM SIERRA DEL AJUSCO, DISTRITO FEDERAL, MEXICO. *The Southwestern Naturalist* **56**, 120–124 (2011).

210. Lemos-Espinal, J. A., Smith, G. R. & Ballinger, R. E. Body temperature and sexual dimorphism of Sceloporus aeneus and Sceloporus palaciosi from Mexico. *Amphibia-Reptilia* **23**, 114–119 (2002).

211. Smith, G. R., Ballinger, R. E. & Congdon, J. D. Thermal ecology of the high-altitude bunch grass lizard, Sceloporus scalaris. *Canadian journal of zoology* **71**, 2152–2155 (1993).

212. Lemos-Espinal, J. A., Smith, G. R. & Ballinger, R. E. Sexual dimorphism and body temperatures of Sceloporus siniferus from Guerrero, Mexico. *Western North American Naturalist* **61**, 498–500 (2001).

213. Gillis, R. Thermal biology of two populations of red-chinned lizards (Sceloporus undulatus erythrocheilus) living in different habitats in southcentral Colorado. *Journal of Herpetology* 18–23 (1991).

214. Lemos-Espinal, J. A., Smith, G. R., Ballinger, R. E. & Smith, H. M. Ecology of Sceloporus undulatus speari (Sauria: Phrynosomatidae) from north-central Chihuahua, México. *Journal of Herpetology* **37**, 722–725 (2003).

215. Parker, S. L. & Andrews, R. M. Incubation temperature and phenotypic traits of Sceloporus undulatus: implications for the northern limits of distribution. *Oecologia* **151**, 218–231 (2007).

216. Angilletta, M. J., Winters, R. S. & Dunham, A. E. Thermal Effects on the Energetics of Lizard Embryos: Implications for Hatchling Phenotypes. *Ecology* **81**, 2957–2968 (2000).

217. Smith, G. R. & Ballinger, R. E. Thermal ecology of Sceloporus virgatus from southeastern Arizona, with comparison to Urosaurus ornatus. *Journal of Herpetology* 65–69 (1994).

218. Foster, M. A., Bissell, K. M., Campa III, H. & Harrison, T. M. The influence of reproductive status on thermal ecology and vegetation use of female Eastern Massasauga rattlesnakes (Sistrurus catenatus catenatus) in southwestern Michigan. *Herpetological Conservation and Biology* **4**, 48–54 (2009).

219. Allen, K. E. & Powell, R. Thermal biology and microhabitat use in Puerto Rican eyespot geckos (Sphaerodactylus macrolepis macrolepis). *Herpetological Conservation and Biology* **9**, 590–600 (2014).

220. Thompson, M. B. Incubation of eggs of tuatara, Sphenodon punctatus. *Journal of Zoology* **222**, 303–318 (1990).

221. Nelson, N. J., Thompson, M. B., Pledger, S., Keall, S. N. & Daugherty, C. H. Egg mass determines hatchling size, and incubation temperature influences post-hatching growth, of tuatara Sphenodon punctatus. *Journal of Zoology* **263**, 77–87 (2004).

222. Besson, A. A., Nelson, N. J., Nottingham, C. M. & Cree, A. Is cool egg incubation temperature a limiting factor for the translocation of tuatara to southern New Zealand? *New Zealand Journal of Ecology* 90–99 (2012).

223. Ma, L. *et al.* Sexual dimorphism, female reproductive characteristics and egg incubation in an oviparous forest skink (Sphenomorphus incognitus) from South China. *Asian Herpetological Research* **9**, 119–128 (2018).

224. Yang, J., Sun, Y.-Y., An, H. & Ji, X. Northern grass lizards (Takydromus septentrionalis) from different populations do not differ in thermal preference and thermal tolerance when acclimated under identical thermal conditions. *J Comp Physiol B* **178**, 343–349 (2008).

225. Xiang, J., Weiguo, D. & Pingyue, S. Body temperature, thermal tolerance and influence of temperature on sprint speed and food assimilation in adult grass lizards, Takydromus septentrionalis. *Journal of Thermal Biology* **21**, 155–161 (1996).

226. Zhihua, L. & Xiang, J. The effects of thermal and hydric environments on incubating eggs and hatchlings of the grass lizard, Takydromus septentrionalis. *Dong wu xue yan jiu= Zoological Research* **19**, 439–445 (1998).

227. Du, W. & Ji, X. Effects of constant and fluctuating temperatures on egg survival and hatchling traits in the northern grass lizard (Takydromus septentrionalis, Lacertidae). *Journal of Experimental Zoology Part A: Comparative Experimental Biology* **305**, 47–54 (2006).

228. Du, W.-G. & Feng, J.-H. Phenotypic effects of thermal mean and fluctuations on embryonic development and hatchling traits in a lacertid lizard, Takydromus septentrionalis. *Journal of Experimental Zoology Part A: Ecological Genetics and Physiology* **309**, 138–146 (2008).

229. Chen, Y.-H., Huang, S.-P., Chang, M.-H. & Tu, M.-C. Thermal Effects on Embryogenesis and Hatchlings of the Grass Lizard Takydromus stejnegeri (Squamata: Lacertidae) and Implications of Their Potential for Limiting Its Altitudinal Distribution in Taiwan. *Zoological Studies* **49**, 374–380 (2010).

230. Zhichong, P. & Xiang, J. The influence of incubation temperature on size, morphology, and locomotor performance of hatchling grass lizards (Takydromus wolteri). *undefined* **21**, 2031–2038 (2001).

231. O'Donnell, R. P. & Arnold, S. J. Evidence for Selection on Thermoregulation: Effects of Temperature on Embryo Mortality in the Garter Snake Thamnophis elegans. *Copeia* **2005**, 930–934 (2005).

232. Charland, M. B. & Gregory, P. T. Movements and habitat use in gravid and nongravid female garter snakes (Colubridae: Thamnophis). *Journal of Zoology* **236**, 543–561 (1995).

233. Gregory, P. T. & Skebo, K. M. Trade-offs between Reproductive Traits and the Influence of Food Intake during Pregnancy in the Garter Snake, Thamnophis elegans. *The American Naturalist* **151**, 477–486 (1998).

234. Wylie, G. D. *et al.* Metals and trace elements in giant garter snakes (Thamnophis gigas) from the Sacramento Valley, California, USA. *Archives of environmental contamination and toxicology* **56**, 577–587 (2009).

235. Wylie, G. D., Casazza, M. L., Halstead, B. J. & Gregory, C. J. Sex, season, and time of day interact to affect body temperatures of the Giant Gartersnake. *Journal of Thermal Biology* **34**, 183–189 (2009).

236. Stewart, G. R. Thermal Ecology of the Garter Snakes Thamnophis sirtalis concinnus (Hallowell) and Thamnophis ordinoides (Baird and Girard). *Herpetologica* **21**, 81–102 (1965).

237. Rosen, P. C. Comparative field study of thermal preferenda in garter snakes (Thamnophis). *Journal of Herpetology* 301–312 (1991).

238. Gibson, A. R. & Falls, B. B. Thermal biology of the common garter snake Thamnophis sirtalis (L.). *Oecologia* **43**, 79–97 (1979).

239. Charland, M. B. Thermal Consequences of Reptilian Viviparity: Thermoregulation in Gravid and Nongravid Garter Snakes (Thamnophis). *Journal of Herpetology* **29**, 383–390 (1995).

240. Rocha, C. F. D., Vrcibradic, D., Menezes, V. A. & Ariani, C. V. Ecology and natural history of the easternmost native lizard species in South America, Trachylepis atlantica (Scincidae), from the Fernando de Noronha Archipelago, Brazil. *Journal of Herpetology* 450–459 (2009).

241. López, P., Civantos, E. & Martín, J. Body temperature regulation in the amphisbaenian Trogonophis wiegmanni. *Canadian Journal of Zoology* **80**, 42–47 (2002).

242. Perez-Mellado, V. & de la Riva, I. Sexual Size Dimorphism and Ecology: The Case of a Tropical Lizard, Tropidurus melanopleurus (Sauria: Tropiduridae). *Copeia* **1993**, 969–976 (1993).
243. Ribeiro, L. B. & Freire, E. M. Thermal ecology and thermoregulatory behaviour of Tropidurus hispidus and T. semitaeniatus in a caatinga area of northeastern Brazil. *The Herpetological Journal* **20**, 201–208 (2010).
244. Kiefer, M. C., Sluys, M. V. & Rocha, C. F. D. Body temperatures of Tropidurus torquatus (Squamata, Tropiduridae) from coastal populations: Do body temperatures vary along their geographic range? *Journal of Thermal Biology* **6**, 449–456 (2005).
245. Ribeiro, L. B., Gomides, S. C., Santos, A. O. & Sousa, B. M. Thermoregulatory behavior of the saxicolous lizard, Tropidurus torquatus (Squamata: Tropiduridae), in a rocky outcrop in Minas Gerais, Brazil. *Herpetological Conservation and Biology* **3**, 63–70 (2008).
246. Gilbert, A. L. & Miles, D. B. Food, temperature and endurance: effects of food deprivation on the thermal sensitivity of physiological performance. *Functional Ecology* **30**, 1790–1799 (2016).
247. Andrews, R. M., Pezaro, N., Doody, J. S., Guarino, F. & Green, B. Oviposition to hatching: Development of Varanus rosenbergi. *Journal of Herpetology* **51**, 396–401 (2017).
248. Lourdais, O., Shine, R., Bonnet, X., Guillon, M. & Naulleau, G. Climate affects embryonic development in a viviparous snake, Vipera aspis. *Oikos* **104**, 551–560 (2004).
249. Ladyman, M., Bonnet, X., Lourdais, O., Bradshaw, D. & Naulleau, G. Gestation, thermoregulation, and metabolism in a viviparous snake, Vipera aspis: evidence for fecundity-independent costs. *Physiological and Biochemical Zoology* **76**, 497–510 (2003).
250. Lu, H.-L., Hu, R.-B. & Ji, X. The variance of incubation temperatures does not affect the phenotype of hatchlings in a colubrid snake, Xenochrophis piscator. *Journal of Thermal Biology* **34**, 138–143 (2009).
251. Xiang, J., Weiguo, D. & Xuefeng, X. Influences of thermal and hydric environments on incubating eggs and resultant hatchlings in a colubrid snake (Xenochrophis piscator). *Dong wu xue bao.[Acta Zoologica Sinica]* **47**, 45–52 (2001).
252. Bell, K., Blomberg, S. & Schwarzkopf, L. Detrimental influence on performance of high temperature incubation in a tropical reptile: is cooler better in the tropics? *Oecologia* **171**, 83–91 (2013).

253. Lemos-Espinal, J. A., Smith, G. R. & Ballinger, R. E. Ecology of Xenosaurus grandis agrenon, a Knob-Scaled Lizard from Oaxaca, México. *Journal of Herpetology* **37**, 192–196 (2003).
254. García-Rico, J., Vega-Pérez, A. D. de la, Smith, G. R., Lemos-Espinal, J. A. & Woolrich-Piña, G. A. Thermal Ecology, Sexual Dimorphism, and Diet of Xenosaurus tzacualtipantecus from Hidalgo, Mexico. *wnan* **75**, 209–217 (2015).
255. Carretero, M. A., Roig, J. M. & Llorente, G. A. Variation in preferred body temperature in an oviparous population of Lacerta (Zootoca) vivipara. *Herpetolog. J.* **15**, 51–55 (2005).
256. Rodríguez-Díaz, T. & Braña, F. Altitudinal variation in egg retention and rates of embryonic development in oviparous Zootoca vivipara fits predictions from the cold-climate model on the evolution of viviparity. *J. Evol. Biol.* **25**, 1877–1887 (2012).
257. Van Damme, R., Bauwens, D. & Verheyen, R. F. Evolutionary Rigidity of Thermal Physiology: The Case of the Cool Temperate Lizard Lacerta vivipara. *Oikos* **57**, 61–67 (1990).
258. Galliard, J.-F. L., Bris, M. L. & Clobert, J. Timing of locomotor impairment and shift in thermal preferences during gravidity in a viviparous lizard. *Functional Ecology* **17**, 877–885 (2003).
259. Van Damme, R., Bauwens, D. & Verheyen, R. F. Selected body temperatures in the lizard Lacerta vivipara: Variation within and between populations. *Journal of Thermal Biology* **11**, 219–222 (1986).
260. Gvoždík, L. To heat or to save time? Thermoregulation in the lizard Zootoca vivipara (Squamata: Lacertidae) in different thermal environments along an altitudinal gradient. *Canadian Journal of Zoology* (2002) doi:10.1139/z02-015.
261. Rodríguez-Díaz, T., González, F., Ji, X. & Braña, F. Effects of incubation temperature on hatchling phenotypes in an oviparous lizard with prolonged egg retention: are the two main hypotheses on the evolution of viviparity compatible? *Zoology (Jena)* **113**, 33–38 (2010).
262. Rodriguez-Diaz, T. & Brana, F. Altitudinal variation in egg retention and rates of embryonic development in oviparous Zootoca vivipara fits predictions from the cold-climate model on the evolution of viviparity. *Journal of Evolutionary Biology* **25**, 1877–1887 (2012).

263. Gvoždík, L. & Castilla, A. A Comparative Study of Preferred Body Temperatures and Critical Thermal Tolerance Limits among Populations of Zootoca vivipara (Squamata: Lacertidae) along an Altitudinal Gradient. (2001) doi:10.2307/1565967.
